# Electrically driven heterostructured far-infrared wire lasers with integrated graphene plasmons

**DOI:** 10.1038/s41565-025-02005-z

**Published:** 2025-10-30

**Authors:** Alessandra Di Gaspare, Sara Ghayeb-Zamharir, Lianhe Li, Edmund H. Linfield, Alexander G. Davies, Jincan Zhang, Osman Balci, Andrea C. Ferrari, Miriam S. Vitiello

**Affiliations:** 1 NEST, CNR-NANO and Scuola Normale Superiore, Pisa, Italy; 2 School of Electronic and Electrical Engineering, University of Leeds, Leeds, UK; 3 Cambridge Graphene Centre, University of Cambridge, Cambridge, UK

**Keywords:** Lasers, LEDs and light sources, Optical properties and devices, Nanophotonics and plasmonics

## Abstract

Photonic technologies that exploit surface plasmons in graphene can offer groundbreaking opportunities for the development of compact and inexpensive active photonic devices, owing to the unique combination of tight field localization, giant optical nonlinearities and electrostatic gating tuning. Here we take advantage of this unique combination of properties to engineer frequency up-converted, electrically driven, single-mode photonic sources in the 9.0–10.5 THz range, with an emission frequency entirely tunable by design. We excite plasmons confined in a multilayer graphene micro-ribbon grating within a distributed-feedback terahertz quantum cascade laser that incorporates a top supercapacitor to tune the graphene Fermi energy, demonstrating third harmonic generation. Our monolithic, electrically driven laser works in the inaccessible Reststrahlen band of its core III–V semiconductor heterostructure and shows a peak power of ~9 μW, laying the foundation of a new generation of plasmonic, nonlinear light-emitting sources.

## Abstract (web summary)

A miniaturized frequency up-converted electrically tunable single-mode photonic source in the 9.0–10.5 THz range is demonstrated.

## Main

Collective charge excitations (plasmons) in single-layer graphene (SLG)^1,2^ present strong similarities to the electromagnetic waves that propagate at the interface between a metal and a dielectric (surface plasmons)^3^. Specifically, they are characterized by a transverse-magnetic polarization^4^ and by an exponentially decaying electric field amplitude in the direction orthogonal to the SLG plane^5^. However, owing to the Dirac-like band profile and locked in-plane electron motion, SLG plasmons have distinctive features that differentiate them from metal surface plasmons^6^, including a tighter field localization and reduced propagation losses^1,2,4,5^. Importantly, the SLG plasmon charge can not only be electrostatically controlled through doping but also by gating^7,8^, so that the behaviour of SLG surface plasmon-based structures can be modified in situ without the need for structural device modifications. At terahertz (THz) frequencies, this can be combined with the graphene’s tunable optical properties^8,9^, offering groundbreaking opportunities for creating compact electrically controllable THz optical components^10^; reconfigurable metamaterials^11–13^; robust, fast (hundred picoseconds response times), cheap and scalable THz frequency photo-detectors^13–15^; and novel light sources^16–18^. However, at THz frequencies, electromagnetic radiation cannot couple directly into bidimensional plasmon excitations and so structures engineered on a subwavelength scale are required, up to 200 times smaller than the wavelength in vacuum^19^—the simplest geometry being a periodic grating of graphene micro-ribbons^20,21^. In this case, the plasmon resonance energy scales as *n*^1/4^ (*n* being the carrier density) for Dirac plasmons in a micro-ribbon array, and as *w*^−2^ (*w* being the ribbon width). The combination of electrical and optical tuning thereby allows the properties of the graphene plasmons to be tailored.

Plasmonic effects can be used to confine THz fields to a subwavelength volume, exploiting the high degree of spatial confinement of graphene plasmons. They can also be used for efficient up-conversion in graphene—also known as harmonic generation (HG). Indeed, the giant SLG nonlinearities (*χ*^(3)^ ≈ 10^−9^ m^2^ V^−^^2^)^22^, observed in the far-infrared, governed by graphene’s intraband carrier dynamics^23^, combined with the inherent ultrafast (picoseconds) carrier dynamics, have enabled high HG at THz frequencies using only moderate fields and at room temperature^24^.

Here we exploit the tight field localization and the giant optical nonlinearities of graphene plasmons to engineer frequency up-converted electrically pumped photonic sources across the 6.0–12.5 THz (24–50 µm wavelength) range. This overcomes the current lack of a spectrally narrowband solid-state-based technology that can access the whole 6.5–12.0 THz frequency range.

## Results and discussion

### Device engineering

**Fig. 1:**
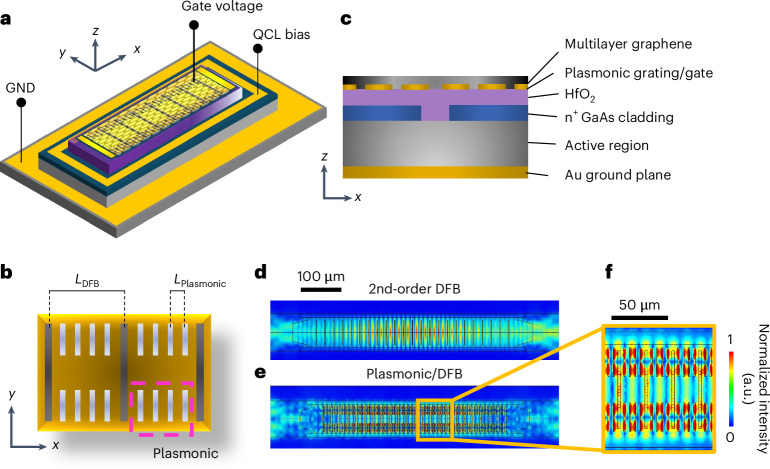
Device concept. **a**–**c**, Schematic perspective view of the hybrid plasmonic QCL design (**a**), featuring a surface-emitting, 2nd-order DFB grating fabricated within the top contact of a double-metal waveguide resonator, with emission centred at ~3.3 THz (*L*_DFB_ = 25.5 μm). The ground (GND) is the bottom part of the QCL double-metal waveguide. The gate voltage is applied on the top. **b**, An overlapping plasmonic MLG/Au ribbon array (pink dashed region in **b**, *L*_Plasmonic_ = 5.1 μm) enhances the intracavity field of the fundamental lasing mode of the DFB cavity. The cross-sectional view of the design (**c**) illustrates the layer sequence. A 30-nm-thick dielectric layer of HfO_2_ between the top contact and the plasmonic/DFB metal contact (purple region in **c**) allows tuning of the Fermi energy E_F_, through the field effect, and hence active control of the MLG optical conductivity, and the resulting field coupling and confinement. The dashed box in **b** marks the top view of the section shown in **c**. **d**–**f**, Electric field distribution of the graphene-integrated QCL cavity simulated using a 3D eigenfrequency study in COMSOL Multiphysics for the standard surface-emitting DFB (that is without plasmonic grating) at the fundamental eigenmode at 3.226 THz (**d**) and including the plasmonic/DFB grating at 3.220 THz (**e**,**f**). Panel **f** shows a magnified area of the plasmonic grating, highlighting the field enhancement in the graphene ribbons at the fundamental TM_00_ mode. a.u., arbitrary units.

For our benchmark device, we engineer a surface-emitting distributed-feedback (DFB)^25^ double-metal^26^ quantum cascade laser (QCL)—a semiconductor heterostructure laser relying on intersubband transitions^27–29^—to include a superimposed multilayer graphene (MLG) plasmonic grating (a graphene ribbon array) and a top capacitor (acting as a gate electrode) (Fig. 1a–c). The DFB resonator is designed with a slit periodicity tuned to match the centre of the gain bandwidth (3.25–3.35 THz) of the selected QCL active region^30,31^; the DFB grating is intended to govern the desired photonic momentum and the frequency of the mode propagating along the longitudinal direction of the resonator bar. The intracavity integrated plasmonic ribbon grating then provides the field enhancement needed for HG, while the top capacitor enables efficient tuning of the graphene Fermi level by electrostatic gating. The result is a double-grating resonator integrated into the top contact of a THz QCL incorporating a second-order DFB grating^32^, for the optimal control of the mode within the laser optical band, overlapping a plasmonic grating, which induces a strong local electric field enhancement needed to drive frequency up-conversion.

We first numerically simulate the resonator to identify the optimal double-grating design. The double-metal waveguide QCL was modelled using COMSOL Multiphysics, with a finite element method solver (Supplementary Section 1). The plasmonic/DFB design was optimized by initially conducting a parametric study on the double-grating resonator unit cell (Supplementary Section 1). This was followed by three-dimensional (3D) simulations of the complete laser structure (Fig. 1a–c), which includes top Cr side absorbers to suppress the lateral cavity modes^33^. Initially, we compared two structures: (i) a standard MLG-coated DFB structure (without the plasmonic grating; Fig. 1d) and (ii) the combined plasmonic/DFB grating device of Fig. 1a–c (Fig. 1e).

The 3D model of the MLG-coated DFB structure shows a fundamental eigenmode at frequency *ν*_DFB_ ≈ 3.226 THz (quality factor *Q*_DFB_ ≈ 71) (Fig. 1d), demonstrating that the integration of the MLG in the slits neither affects the resonator modes nor prevents laser action. The plasmonic grating is integrated into the top contact of the DFB resonator (Fig. 1a–c). To mitigate the induced increase in the total losses, we only patterned the MLG ribbon array towards the edges of the top contact region, that is, leaving the central region untouched (Fig. 1b). The MLG plasmonic grating leads to an eigenmode at *ν*_pl_ ≈ 3.220 THz (Fig. 1e,f), with a field distribution resembling that of the fundamental mode, aside from a slightly different field lobe distribution along the *y* axis. Importantly, we observe a substantial optical coupling inside the plasmonic slits between the intracavity field and the MLG (Fig. 1f). The *Q* value of the fundamental eigenmode *ν*_f1_, *Q*_f1_ ≈ 29, is lower than that of the MLG-coated DFB, as a consequence of the increased optical losses introduced by the plasmonic slits. However, the electric field amplitude in the MLG is amplified, on average, by a factor *A*_ave_ ≈ 2.5. This value is obtained by calculating the average electric field amplitude across the entire top contact surface. In the central region of the plasmonic grating, where the enhancement of the intracavity field is maximum, the amplification is increased by almost 2 orders of magnitude, *A*_peak_ ≈ 10^2^.

### Simulation model of the frequency up-conversion process

To evaluate the role of the electric field enhancement on the HG process occurring in the top surface grating (MLG ribbons), and to estimate the expected HG conversion efficiency (CE), we follow the approach of ref. ^34^. This method allows the third harmonic generation (THG) efficiency to be extracted as an output parameter directly from the simulations, by setting up the equations for the third harmonic generated field in the software module. The method assumes that the MLG is a nonlinear surface current generator (Supplementary Section 2) and computes the CE (Supplementary Sections 3 and 4) of the THG process by solving the Maxwell equations at the third harmonic frequency, assuming a quasi-continuous wave excitation with an average 300 mW input power.

**Fig. 2:**
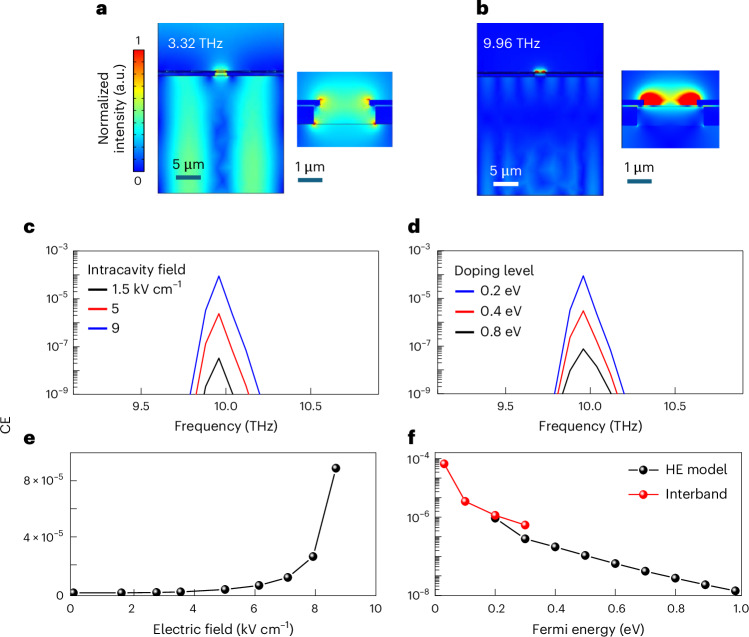
Simulation of the on-chip frequency up-conversion process. **a**,**b**, Cross-sectional views of the surface electric field distribution calculated at the fundamental mode at ~3.32 THz (**a**) and the third harmonic frequency of ~9.96 THz (**b**), for the DFB/plasmonic resonator coated with three layers of graphene, simulated by employing a surface current density numerical model to run two-dimensional simulations with the frequency domain module of COMSOL Multiphysics. The right sides of **a** and **b** show the magnified views of the electric field in the DFB slit region, respectively; the intensity of the colour map in **b** is a ×300 magnification of the intensity of that in **a**. The length of the unit cell along the *x* axis matches the periodicity of the DFB array (25.5 µm). The GaAs active region was modelled as a constant refractive index material in the THz range (*n* = 3.6), with the DFB grating realized by etching an air hole of 0.7 µm depth and 2.5 µm width inside the doped layer. The refractive index of HfO_2_, as a gate dielectric with 30 nm thickness, was set at 4.24. An array of slits with periodicity 1/5 of the DFB periodicity were incorporated into the metal and then coated with three layers of graphene, thus realizing graphene micro-ribbons embedded in the top metal, here modelled as a perfect electric conductor. Perfectly matched layer and perfect electric conductor boundary conditions were selected for the top and bottomdirections along *z* (see Fig. 1c), respectively, while periodic boundary conditions were set along *x* (see Fig. 1c). All the simulations were performed using a single periodic excitation port, illuminating the top surface. To mimic the intracavity field intensity driving the nonlinear response in the plasmonic array, the input port power was set to 300 mW, concentrated over the area of a single DFB slit. **c**,**d**, TH CE as a function of frequency for different intracavity fields (**c**) and values of *E*_F_ (**d**). Other graphene parameters were electron mobility 1,400 cm^2^ V^−1^ s^−1^ and scattering time 23 fs. **e**, CE as a function of the intracavity electric field, assuming *E*_F_ = 200 meV. **f**, CE as a function of *E*_F_, assuming an intracavity field of ~8.6 kV cm^−1^, calculated using a 3rd-order nonlinear model based on hot electron (HE) intraband absorptions (black) at *E*_F_ ≥ 200 meV, and interband multiphoton absorptions (red) at *E*_F_ ≤ 200 meV, respectively. *E*_F_ is assumed equal to 200 meV in **a**–**c** and **e**; the intracavity field is assumed equal to ~8.6 kV cm^−1^ in **f**. a.u., arbitrary units.

The calculated surface electric field distribution at the fundamental mode ~3.32 THz (Fig. 2a) and at the TH frequency ~9.96 THz (Fig. 2b) for the double-grating QCL with a 3-layer graphene plasmonic ribbon grating shows that, at the TH frequency (Fig. 2b), the field distribution mimics that retrieved at the fundamental mode, with a field intensity more than 2 orders of magnitude lower. Figure 2c,d shows the calculated CE as a function of frequency, at different intracavity fields and MLG Fermi energies, *E*_F_, respectively. At three times the fundamental frequency, the predicted CE is ~10^−4^ for a moderate value of *E*_F_ ~200 meV. The calculated decrease in the TH peak intensities for increasing *E*_F_ (Fig. 2f) differs from previous reports^35^ on TH CEs in doped graphene, where the nonlinear response is stronger in highly conductive Dirac systems^35^. This behaviour stems from the different excitation dynamics provided by the quasi-continuous wave laser, adopted in the present case.

At the Dirac point, a larger CE is predicted to be achieved (Fig. 2f). However, the very low Fermi energies (<50 meV) required to achieve this condition are extremely difficult to realize in large-area graphene, even with a very efficient gate tuning. A more reliable comparison is then with the CE calculated at the Fermi level *E*_F_ ≈ 200, that is, the value set after optimizing our simulations. We set the maximum intracavity power at 0.3 W (≈9 kV cm^−1^ intracavity field) (Fig. 2e) in our analysis, because our model loses validity at higher powers as the electronic temperature becomes higher than the Fermi temperature. Under the latter conditions, the smearing-out of the carrier distribution opens a channel for interband transitions^36^, involving multiphoton absorption^37^ beyond Pauli blocking, that must be taken into account when evaluating the CE (Fig. 2f). It is worth mentioning that at 3*ν*_0_, Reststrahlen band phonons play no role in the TH up-conversion process, since, in our geometry, the nonlinear effects inducing THG take place in a very confined volume (<0.1 μm^3^) around the plasmonic ribbon surface, and so they are spatially separated by the absorbing medium (Supplementary Section 4).

### Demonstration of efficient THG

**Fig. 3:**
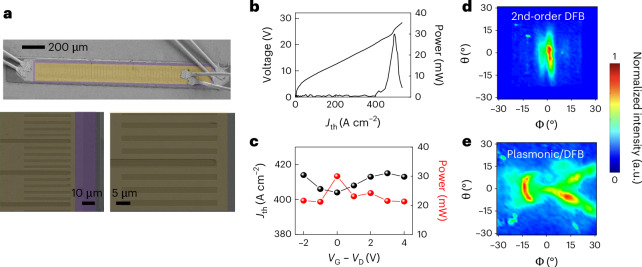
Device fabrication, electrical and optical characterization. **a**, Scanning electron microscope images of a prototypical fabricated device, showing the top DFB/plasmonic grating (yellow, false colour) and the gate oxide area (purple). The grating comprises a plasmonic ribbon of width *W*_pl_ ≈ 2 µm and a ribbon spacing of 1/5 of the periodicity of the DFB array. At such a ribbon width, the plasmonic resonance of the MLG /Au ribbon grating, on the lateral edges of the top metal, matches the DFB mode. **b**, Light–current density–voltage (*L–J–V*) characteristics measured on a 1.5 × 0.2 mm-sized plasmonic/DFB QCL bar, when driving the QCL in pulsed mode with a pulse width of 1 µs (duty cycle 5%) at 15 K. **c**, QCL threshold current density (left axis, black) and emission power (right axis, red), as a function of the gate voltage. **d**,**e**, Far-field profiles measured for a standard DFB (**d**) and a plasmonic/DFB (**e**) QCL, realized on the same active region and with the same DFB array geometry. The far-field profiles were measured under the same conditions of **b**, at a driving current corresponding to the peak optical power, while raster scanning a pyroelectric detector placed at ~5 cm from the laser surface, in the plane parallel to the laser surface, and projecting the two-dimensional signal onto a spherical surface, identified by the azimuthal (Φ) and the polar (θ) angles. a.u., arbitrary units.

We then fabricate a set of surface-emitting double-metal QCLs following the device schematics in Fig. 1a–c, using a high-power THz QCL delivering 2.5 W peak power^30,31^ (Supplementary Sections 5 and 6). The DFB grating (Fig. 3a) was designed as a linear array of 2.5-µm-wide, 800-nm-deep slits in the top metal/doped layer. After patterning the top DFB grating, using optical lithography, followed by the removal of the doped GaAs from the slits, we cover the top cladding layer with an ~30-nm-thick layer of HfO_2_ using atomic layer deposition. This enables field-effect gate coupling of the MLG in the top emitting surface and the electronic control of *E*_F_. We then used electron beam lithography to pattern the top plasmonic grating by aligning the DFB/plasmonic grating pattern with the underlying DFB slits. The MLG transfer was performed by a poly(methyl methacrylate) (PMMA)-assisted wet method^38^, using sequential SLG transfers (Methods and Supplementary Section 7), placing three graphene layers on the QCL devices.

We fabricated seven devices, each demonstrating a consistent behaviour (Supplementary Section 8). The voltage–current density–light characteristic (Fig. 3b for one typical device) shows a maximum peak optical power of ~30 mW. The presence of the static gate electrode coupled with the laser top contact (Fig. 3a) has only a marginal impact on both the emitted optical power and laser threshold, with the latter varying by ≤10 A cm^−^^2^ at the highest gate voltage (Fig. 3c). However, the gate bias (*V*_G_) provides an efficient tool to change the MLG *E*_F_, and enables tuning of the CE, as predicted by the theoretical model (Fig. 2d,f). The comparison between the far-field intensity profile of the integrated laser (Fig. 3e) and that of a standard surface-emitting 2nd-order DFB QCL (Fig. 3d and Supplementary Section 9) shows that, while the typical single lobe profile with ~≤10° divergence^25,39^ is obtained in the latter case, the plasmonic grating induces two side lobes with an ~15° angular broadening. This is understood by considering the field coupling of the two series of plasmonic slits, defined along the two edges of the top contact. Raman spectroscopy was used to confirm the MLG quality following transfer on the QCL device^40^ (Supplementary Section 7).

To verify the occurrence of the expected third-order frequency up-conversion process, we mounted the devices in a Fourier transform infrared (FTIR) spectrometer under vacuum and collected spectra in step-scan mode over long acquisition times. To isolate the third harmonic terms at 3*ν*_0_ from the fundamental 2nd-order DFB lasing mode at *ν*_0_, we used a high-pass thallium filter (Crystan) positioned in front of a Si-bolometer detector. This suppresses >95% of the power <6 THz (ref. ^41^), with a transmittance ≥50% in the 6–7 THz range, and ≥70% at frequencies >8 THz (Supplementary Section 10).

**Fig. 4:**
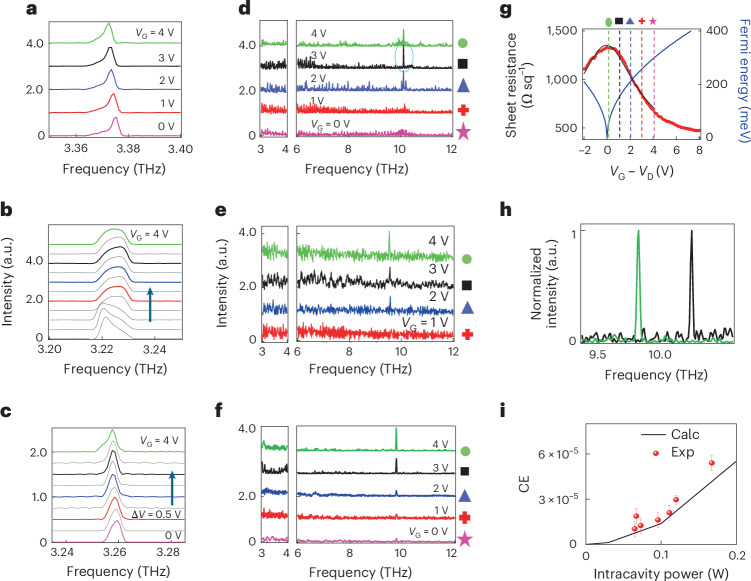
THG and CE. **a**–**c**, Stacked normalized FTIR emission spectra acquired, while driving the QCL with a current corresponding to the peak optical power, as a function of the gate voltage *V*_G_, for three QCLs with differing DFB grating pitches (**a**–**c**), and fabricated with an optimized dry etching fabrication protocol (**c**). The spectra were measured in rapid-scan mode, under vacuum, using a helium-cooled Si bolometer (IRLabs), with a spectral resolution of 0.075 cm^−1^. The QCL in **b** is initially emitting at 3.222 THz; this frequency initially red-shifts with tuning of the graphene Fermi level, but the QCL then mode hops at 3.228 THz before red-shifting up to 3.225 THz as *V*_G_ increases to 4 V. At zero gate bias (*V*_G_), *ν*_0_ = 3.375 THz (**a**), *ν*_0_ = 3.221 THz (**b**) and *ν*_0_ = 3.262 THz (**c**). **d**–**f**, Stacked FTIR emission spectra acquired in step-scan mode, under the same experimental conditions as **a**–**c** with a spectral resolution of 1 cm^−1^, and filtering out the QCL lasing modes with a Ta high-pass filter (cut-off ~7 THz), for the three QCLs in **a** (**d**), **b** (**e**) and **c** (**f**). The step-scan signal was retrieved with a lock-in amplifier (Stanford Instruments), synchronized with an amplitude-modulated signal of 317 Hz, which was used to modulate the pulsed bias driving the QCL. The left sides of **d**–**f** show the step-scan spectra measured in the range of emission of the DFB QCL. In **a**–**c**, the traces are acquired at intermediate (grey) and specific gate voltages: *V*_G_ = +4 V (green), +3 V (black), +2 V (blue), +1 V (red) and 0 V (pink, **a** and **c**), that are marked by symbols in **d**–**f**; in these samples, we assume that the minimum conductivity point is at *V*_G_ = *V*_D_ ≈ 4.5 V. **g**, Sheet resistance modulation as a function of the gate voltage applied to an ideal, microscopic MLG graphene FET, realized with the same gate architecture used for the top-QCL supercapacitor (left axis, red dots) to tune *E*_F._ The black curve is the fit to the experimental data. The *V*_G_ dependence of *E*_F_ (blue line, right axis) is extracted from ${E}_{{\rm{F}}}=\hbar {v}_{{\rm{F}}}\sqrt{{\rm{\pi }}n\left({V}_{{\rm{G}}}\right)}$. **h**, Expanded view of the third harmonic signal measured at *V*_G_ = +3 V on the laser of **a**, highlighting an S/N ratio of about 20 (black curve), and at *V*_G_ = +4 V on the optimized laser of **c**, highlighting an S/N ratio of about 60 (green curve). **i**, Comparison between the calculated CE (Calc, black line) and the CEs retrieved experimentally (Exp, red dots) across a set of seven devices, the last one (intracavity power 0.17 W) belonging to the new batch, each having different intracavity powers, displayed as a function of the corresponding intracavity power (Supplementary Section 8). Data are presented as mean values ± standard error of the mean (s.e.m.) bar, calculated following the relative error method, namely considering the s.e.m. of the signal amplitude of the third harmonic signal, as extracted from the interferogram trace, according to the method illustrated in Supplementary Section 8, and assigning a corresponding linearly proportional value to the CE error bar. a.u., arbitrary units.

Figure 4a–f plots the rapid-scan unfiltered (Fig. 4a–c) spectra, and step-scan filtered (Fig. 4d–f) emission spectra, at different gate voltages, measured on three devices. The first two devices of Fig. 4a,b were fabricated with a 2nd-order DFB pitch slightly detuned in frequency, within the 0.5-GHz-wide bandwidth of the QCL (Supplementary Section 6). The third device (Fig. 4c) belongs to a different fabrication batch, realized with an improved fabrication process, in which the DFB slits have been dry etched to engineer smoother sidewalls and a flat surface, preventing possible under-etching effects. At zero *V*_G_, the measured *ν*_0_ is in agreement with the DFB grating design, in which photons are backscattered if the condition *k*_p_ = 2*k*_B_ – *k*_p_ is fulfilled, with *k*_B_ and *k*_p_ being the wavevector of the Bragg peak and of the photon in the waveguide, respectively. *ν*_0_ consistently tunes with *V*_G_, red-shifting by ~15 GHz V^−1^ for the sample of Fig. 4a, and by ~5 GHz V^−1^ for the sample of Fig. 4c, as *V*_G_ approaches the MLG minimum conductivity point. In our integrated structures, it occurs at *V*_G_ = +5 V, close to *V*_D_, the Dirac voltage measured on an ideal MLG field-effect transistor (FET) with an identical gate architecture (Fig. 4g). A visible mode hop is noticeable in Fig. 4b, which is the cause of the different retrieved lineshapes and frequency shift. The observed trend is likely related to the refractive index variations (and corresponding emission frequency variations) owing to gain change with the pump current that can be estimated via the linewidth enhancement factor^42^. The pump current is indeed affected by the conductivity of the material layer in the ribbon apertures^18^.

In all three cases (Fig. 4d–f), a well-defined peak emerges above the noise level at 3*ν*_0_ = 10.1 THz (Fig. 4d), 9.66 THz (Fig. 4e) and 9.78 THz (Fig. 4f), with a signal-to-noise ratio ranging from S/N ≈ 5 to S/N ≈ 60 (Fig. 4h) for *V*_G_ ≳ *V*_D_ −2 V to V_G_ ≈ V_D_. These peaks correspond to the expected THG process in the MLG plasmonic grating. At *V*_G_ < V_D_ −2 V, the THG peak is still visible but consistently decreases in amplitude and then disappears below the noise level. The same behaviour was confirmed in most of the devices tested (Supplementary Section 8), and was unequivocally ascribed to the MLG since any possible frequency up-conversion process, activated by nonlinearities in the AR or in the HfO_2_ dielectric layer, was excluded (Supplementary Sections 12 and 14).

### Experimental tuning of the CE

To corroborate our observations, we measured the gate modulation on an ideal FET, fabricated by using the same dielectric layer grown on doped GaAs, with a microscopic (10 × 10 µm^2^) MLG channel. The resistivity (red dots, Fig. 4g) is then fit with $$R\left({V}_{{\rm{G}}}\right)={\mu e{n}_{\mathrm{Tot}}\left({V}_{{\rm{G}}}\right)}^{-1}={\left[\mu e\sqrt{{n}_{0}^{2}+{n}^{2}\left({V}_{{\rm{G}}}\right)}\right]}^{-1}$$$$={\left[\mu e\sqrt{{n}_{0}^{2}+{\left(\frac{{C}_{\mathrm{EG}}}{e}\right)}^{2}{\left({V}_{{\rm{G}}}-{V}_{{\rm{D}}}\right)}^{2}}\right]}^{-1}$$.

From the fit, we extract a mobility *µ* ≈ 1,400 cm^2^ V^−1^ s^−1^, a capacitance *C*_EG_ ≈ 215 nF cm^−2^, a residual carrier density *n*_0_ ≈ 3.28 × 10^12^ cm^−2^ and *V*_D_ ≈ 4.4 V. The maximum sheet resistance, retrieved close to the charge neutrality point, was ~1.4 kΩ sq^−1^, indicating a non-negligible background doping, which is not modulated by the gate voltage. The *E*_F_ dependence of *V*_G_ is extracted from ${E}_{{\rm{F}}}=\hbar {v}_{{\rm{F}}}\sqrt{{\rm{\pi }}n\left({V}_{{\rm{G}}}\right)}$. We assume that the gate voltage range probed on the QCLs roughly corresponds to *E*_F_ ranging from 50 meV to a maximum Δ*E*_F_ ≈ 300 meV (Fig. 4g). This assumption is explained as follows. In the large-area (1.2 × 0.12 mm^2^) MLG FET embedded in the QCL, it is difficult to reach the charge neutrality point (*V*_D_) owing to an inhomogeneous *E*_F_ over the sample surface, which prevents carrier depletion at a single gate voltage. The more likely scenario is the achievement of a minimum conductivity point at *V*_G_ corresponding to a low *E*_F_ that we assume to be ~50 meV, as also confirmed by Raman spectroscopy (Supplementary Section 7).

Through the optimized fabrication procedure used for the device of Fig. 4c,f, we could noticeably improve the S/N ratio at the third harmonic frequency, as can be seen from Fig. 4h, showing a direct comparison between the most intense peak retrieved in Fig. 4d (black trace in Fig. 4h) and the best normalized peak collected in the optimized emitter (green curve in Fig. 4h). It is worth mentioning that while a visible peak at the third harmonic frequency is retrieved, the spectra in the frequency range around 6.5 THz, where a possible peak owing to second harmonic emission should appear, are noticeably flat (Supplementary Section 13).

We then estimated the experimental THG CE, by considering the amplitude ratio between the intensities of the frequency up-converted and incident (intracavity) light beams. The intensity of the THG signal is retrieved directly from the interferogram that encodes the signal measured by the lock-in amplifier during the step-scan acquisition, and with a Ta filter in place that removes any optical signal below 4 THz (cut-off frequency 7 THz; Supplementary Section 10). The incident (intracavity) light is extracted from the interferogram collected without the filter, and then normalized accounting for the device internal quantum efficiency (60%)^43^, assuming ~50% light absorption through the MLG^44,45^. The procedure is discussed in detail in Supplementary Section 11 and follows the procedures described in ref. ^41^.

The intracavity power of each device (horizontal axis Fig. 4i) was quantified by considering the actual optical output power, measured with a calibrated Thomas Keating thermal detector placed in front of the cryostat window (Fig. 3b), and then normalized by the internal quantum efficiency (60%) and MLG absorption losses (50%). For the entire set of fabricated samples, the CE values range from ~1 to 5.4 × 10^−5^ (Fig. 4i), matching our simulations. Assuming those values, we obtain a maximum third harmonic peak power, that is, at 9–10 THz, of 9.0 µW (average power of 450 nW).

We also validated this estimate of the emitted power at the third harmonic, following the procedure that we used in a previous work (Fig. 4b in ref. ^41^) for the QCL shown in Fig. 4a. In this case, we isolated the up-converted signal, positioning an 7 THz high-pass Ta filter along the optical path in front of the window of a Ge bolometer (QMC), and detected directly the signal emitted by the integrated laser with a lock-in amplifier, referenced to the same signal used to amplitude-modulate the QCL, driven at a current corresponding to the peak power. Considering the detector responsivity (3.5 kV W^−1^), the lock-in signal gave an optical power output at the third harmonic of 210 nW average power (4.1 µW peak power), slightly larger but comparable with the number retrieved from the procedure described above.

## Conclusion

The demonstration of frequency up-conversion, using electrically controllable graphene plasmons integrated into a semiconductor heterostructure laser, opens up a breadth of new device possibilities, including the tailored design of solid-state sources for the, normally inaccessible, 6.5–12.0 THz frequency range. Far-infrared spectroscopy of a large number of rotational and roto-vibrational transitions of light molecules and free radicals can be easily measured with the achieved 400 nW of average power levels, as already shown at lower frequencies (3 THz) with only 100 nW of optical power^46^. Further exemplar applications include the studies of complex liquids, such as water in the far-intrared range, where strong absorptions are present, or mapping the intricacies of protein function in amino acids such as dipeptides and tripeptides, in a frequency range in which they show resonances^47^. Such applications have not been extensively investigated so far owing to the lack of appropriate sources, but would offer new insights into low-frequency intermolecular motions. This could provide vital information on, for example, the complex interaction of water molecules with atmospheric gases^48^, their role in meso-structures (proteins and charge groups) of biological organisms^49^, and enable fast detection of complex amino acids^50^. The developed sources could also be exploited in detectorless near-field scattering-type scanning near-field optical microscopy systems for quantum nanoscopy applications in the unexplored 24–50 µm range where many plasmonic, phononic and magnetic phenomena of contemporary interest occur^51^. Indeed, by using re-injection of the up-converted light back into the QCL cavity, through the plasmonic grating, the QCL itself can be used as a transducer to reconstruct the near-field maps at multiple frequencies, including the high up-converted THz frequencies.

Technological strategies to increase the output power are likely to lead to wider uptake of the technology. These include (i) engineering the DFB resonator to comprise hybrid dielectric/metal side absorbers to suppress undesired lateral higher-order modes^39^; (ii) more advanced and refined cavity designs that can substantially enhance the CE, for example, adopting plasmonic designs that offer better field enhancement than simple ribbon geometries^52^; and (iii) designing a plasmonic lattice decoupled from the top surface of the laser device, or engineering a resonant cavity at a quarter wavelength thickness to increase further the local electric field density. The latter would lead to minimal changes in the optical losses but result in at least a one order-of-magnitude increase in extraction efficiencies. Those improvements can potentially also open up opportunities for the quantum control of condensed matter systems and their application in quantum computing architectures^53,54^.

## Methods

### MLG preparation and integration onto the QCL chip

The large-area SLG was synthesized on a copper substrate using chemical vapour deposition (CVD)^55^. The SLG on Cu (1 cm × 1 cm) was then coated with a 20-nm-thick layer of polymer (A4-950K PMMA polymer) and cured at 90 °C for 60 s. The graphene film from the bottom surface of the CVD sample was then removed using oxygen plasma reactive ion etching, and the sample immersed, floating and face-up, in a solution of 1 g of ammonium persulfate and 40 ml of deionized water to etch the copper substrate, and remove completely the Cu sacrificial substrate. Once the copper etching was complete, the PMMA-SLG film was transferred into a beaker of deionized water and then scooped up with a second copper–graphene CVD sample, to form a two-layer graphene stack. This sample was left to dry completely. The copper of the bilayer graphene was then etched with the same technique. This process was repeated sequentially until the desired MLG thickness was reached, in this case three layers. The multi-stack was then transferred onto the top of the laser chip, left to dry and finally soaked in acetone to clean the surface and remove the PMMA.

### Reporting summary

Further information on research design is available in the Nature Portfolio Reporting Summary linked to this article.

## Online content

Any methods, additional references, Nature Portfolio reporting summaries, source data, extended data, supplementary information, acknowledgements, peer review information; details of author contributions and competing interests; and statements of data and code availability are available at 10.1038/s41565-025-02005-z.

## Supplementary information

Supplementary Information Supplementary Sections 1–14 and Figs. 1–14. Section 1. Numerical simulations of the device. Section 2. Numerical model for third harmonic generation in the electrically pumped laser structure. Section 3. Estimation of the conversion efficiency. Section 4. Role of the GaAs Reststrahlen band on the CE. Section 5. Device fabrication. Section 6. High-power QCL design. Section 7. Micro-Raman spectroscopy on the MLG. Section 8. Summary of QCL performances. Section 9. Second-order DFB quantum cascade laser. Section 10. High-pass Ta filter transmittance. Section 11. Experimental procedure to extract the collection efficiency. Section 12. Double-grating surface-emitting QCL without MLG. Section 13. Second harmonic generation in MLG-integrated QCLs. Section 14. Lasers fabricated without HfO_2_.

Reporting Summary

## Data Availability

The data presented in this study are available on reasonable request from the corresponding author.

## Code availability

The relevant computer codes supporting this study are available from the authors upon reasonable request.
