# Supplementary material for: Electrically driven heterostructured far-infrared wire lasers with integrated graphene plasmons
Source: Nat Nanotechnol. 2025 Oct 30;20(11):1611–7. doi: 10.1038/s41565-025-02005-z (PMC12623234; doi:10.1038/s41565-025-02005-z)
Supplement: Supplementary file 1 — Supplementary Sections 1–14 and Figs. 1–14. Section 1. Numerical simulations of the device. Section 2. Numerical model for third harmonic generation in the electrically pumped laser structure. Section 3. Estimation of the conversion efficiency. Section 4. Role of the GaAs Reststrahlen band on the CE. Section 5. Device fabrication. Section 6. High-power QCL design. Section 7. Micro-Raman spectroscopy on the MLG. Section 8. Summary of QCL performances. Section 9. Second-order DFB quantum cascade laser. Section 10. High-pass Ta filter transmittance. Section 11. Experimental procedure to extract the collection efficiency. Section 12. Double-grating surface-emitting QCL without MLG. Section 13. Second harmonic generation in MLG-integrated QCLs. Section 14. Lasers fabricated without HfO2. [file 41565_2025_2005_MOESM1_ESM.pdf]

nature nanotechnology

Supplementary information https://doi.org/10.1038/s41565-025-02005-z

Electrically driven heterostructured far-infrared wire lasers with integrated graphene plasmons

In the format provided by the authors and unedited

Supplementary Information

Contents

S1. Numerical simulations of the device
S2. Numerical model for third-harmonic generation in the electrically pumped laser structure
S3. Estimation of the conversion efficiency
S4. Role of the GaAs *Reststrahlenband* on the CE
S5. Device fabrication
S6. High power QCL design
S7. Micro-Raman Spectroscopy on the MLG
S8. Summary of QCL performances
S9. 2nd order DFB quantum cascade laser
S10. High-pass Ta filter transmittance
S11. Experimental procedure to extract the collection efficiency
S12. Double-grating surface emitting QCL without MLG
S13. Second harmonic generation in MLG-integrated QCLs
S14. - Lasers fabricated without HfO_2

S1. Numerical simulations of the device

To design our engineered laser, we first performed numerical simulations using COMSOL Multiphysics. The core structure includes a multilayer graphene (MLG) plasmonic grating overlapped with 2nd-order distributed feedback (DFB) grating on the top surface of a double-metal terahertz (THz) frequency quantum cascade laser (QCL). The 2nd-order DFB selects the main mode within the laser bandwidth, whilst the plasmonic grating provides the field enhancement needed for frequency up-conversion.

The design was optimized by conducting a parametric study on the DFB resonator unit cell, for which the 2D-dimensional (2D) structure is shown in Supplementary Fig.1a, with a size that corresponds to the Bragg lattice periodicity, L_{DFB}=25.5 μm. The simulation unit cell comprises a double-metal waveguide, embedding the GaAs/AlGaAs heterostructure, which is set to a thickness of 23 μm, matching the height of the QCL active region (AR) and cladding layers. The bottom metal is modelled as perfect electric conductor (PEC), with the top contact metal modelled as a Drude metal. Below the top metal, a 700 nm n-doped cladding (3.5×10^{18}cm^{-3}) layer is removed only from the 2.5 μm slit area. The optical constants of the n-doped layer are calculated within the Drude-Lorenz model, with the free-carrier conductivity determined from the doping concentration of ~5×10^{18} cm^{-3} and scattering time τ=325 fs, and with a transverse optical phonon absorption at 8 THz.[1] The AR is modelled as a loss-less dielectric (refractive index imaginary part = 0) with n (GaAs) = 3.6. Our initial

design rationale was to embed a plasmonic grating in the Au contact of the DFB resonator. In principle, to maximize the field amplification in the ribbon grating, the most convenient approach was to engineer a structure in which the two resonances would coincide.

The DFB resonant frequency scales as $\nu_{DFB} \sim cn_{DFB}/L_{DFB}$, with L_{DFB} being the DFB period, and n_{DDFB} the refractive index of the waveguide core layer. The plasmonic mode resonant frequency n_{plasm} is then predicted to scale as[2]:

$$\nu_{plasm} = \left(\frac{e^2 v_F \sqrt{\pi}}{2\hbar} * \frac{\sqrt{n_{2D}}}{w_{plasm}} * \frac{1}{\varepsilon_0 \varepsilon_{plasm} ln[2 \csc(\pi w_{plasm}/L_{plams})]} \right)^{\frac{1}{2}} \qquad \text{(S1)}$$

Here n_{2D} is the graphene doping density, ε_{plasm} is the dielectric constant of the medium hosting the graphene, w_{plasm} is the graphene ribbon width, and L_{plasm} is the periodicity of the plasmonic grating. Therefore, $\nu_{plasm} \sim 1/\mathrm{nplasm} \sqrt{w_{plasm}}$, as for isolated graphene ribbons, where $n_{plasm} = \sqrt{\varepsilon_{plasm}}$.

From this analysis, it can be seen that the DFB resonance relies inherently on the pitch of the Bragg lattice, to ensure the momentum outcoupling with the free-space plane wave irradiated by the top surface of the laser. In contrast, the plasmonic resonance stems mainly from the isolated ribbon, and is only weakly affected by the array periodicity, aside from a weak logarithmic dependence on the grating duty cycle w_{plasm}/L_{plasm}. We thus concluded that the strategy of engineering the same resonance into a single grating was not the best choice. Instead, overlapping two independently designed gratings, i.e. the plasmonic and DFB gratings, into the top metal was an optimal solution. On this basis, we engineered the plasmonic grating (ribbon) as a five-slit grating aligned so that the DFB slit is at the centre of the unit cell, with a periodicity L_{plasm}=5.1 µm, and a fixed ratio between the DFB and the plasmonic grating periodicities given by L_{DFB}/L_{plasm}=5.

The integration of MLG into the resonator is numerically realized by defining the MLG as a transition boundary condition with assigned *n, k* optical constants and thickness $t_{MLG} = N \times 0.35$ nm, where N is the number of graphene layers in the stack. The *n, k* are calculated by following the method of Ref[3], i.e. assuming that the optical response of graphene in the THz range originates from the intraband absorption, and calculating the MLG conductivity according to the Drude model[4,5]:

$$\sigma_{Drude}(\omega) = N\sigma_{DC} \frac{1}{1 - i\omega\tau} \qquad \text{(S2)}$$

where τ is the scattering time, $\sigma_{DC} = \frac{2e^2}{h} |k_F| v_F \tau$ is the static conductivity of Dirac fermions in graphene, v_F = 1 ×10^6 m/s is the Fermi velocity, k_F is the Fermi momentum defined as $k_F = \sqrt{\pi n}$, and n is the 2D electron gas density of the Dirac system. τ is related to the mobility μ through the relation: $\tau = \frac{\mu E_F}{e v_F}$. The three-dimensional conductivity in graphene can be written as $\sigma_{MLG} =$

$\frac{\sigma_{Drude}(\omega)}{t_{MLG}}$. The complex dielectric function for MLG on a dielectric substrate with refractive index n_{sub} is:

$$\varepsilon_{MLG}(\omega) = 1 + \frac{i\sigma_{MLG}(\omega)}{(n_{sub}+1)\varepsilon_0\omega} \tag{S3}$$

The real and imaginary part of the complex refractive index are then defined as:

$$n_{SLG} = Re(\sqrt{\varepsilon_{MLG}(\omega)}) \tag{S4}$$

$$k_{SLG} = Im(\sqrt{\varepsilon_{MLG}(\omega)}) \tag{S5}$$

The conductivity $\sigma_{Drude}(\omega)$ in eq.S2 can be alternatively expressed as:

$$\sigma_{Drude}(\omega) = N\frac{-iD_0}{\pi}\frac{1}{(2\pi\nu+i\Gamma_0)} \tag{S6}$$

where $\omega = 2\pi\nu$, $\Gamma_0 = \tau^{-1}$ is the scattering rate, and $D_0 = E_F e^2/\hbar^2$ is the linear Drude weight.

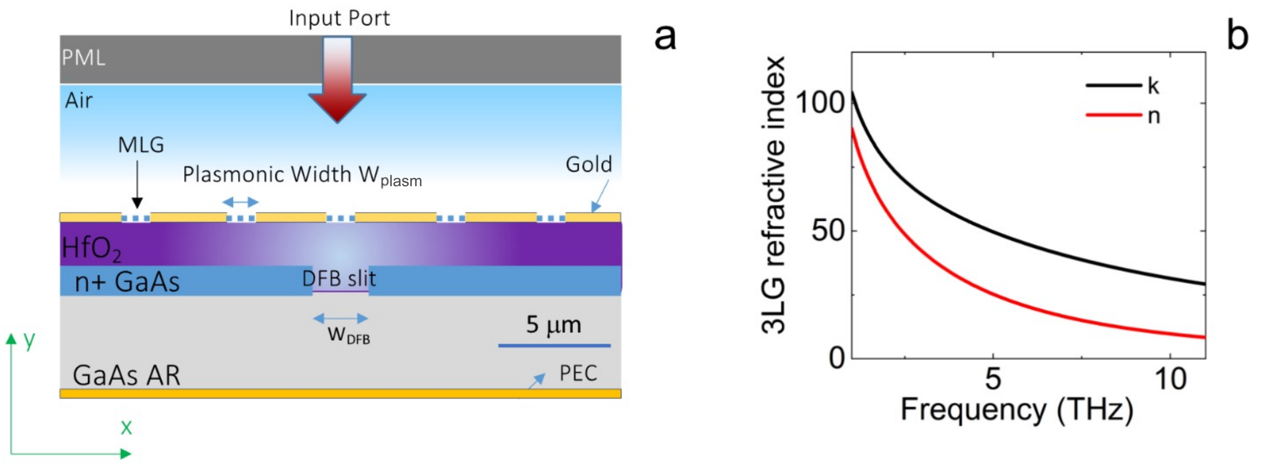

Supplementary Figure 1. (a) Schematic representation of the unit cell resonator design for two-dimensional (2D) simulations using the frequency domain module of COMSOL Multiphysics. The length of the unit cell along x matches the periodicity of the distributed feedback (DFB) array (25.5 µm). The GaAs/AlGaAs active region was modelled as a constant refractive index material in the THz range (n=3.6). The DFB grating was realized by including one air hole with 0.7 um depth and 2.5 um width inside the doped layer. The refractive index of HfO_2, as a gate dielectric with 100 nm thickness, was 4.24. An array of plasmonic gratings with a periodicity of 1/5 of the DFB periodicity was integrated into the gold top contact layer. The gold top contact was defined by the Drude model with $\epsilon = \epsilon_\infty - \frac{f_p^2}{f(f-i\gamma)}$ where $f_p = 2069$ THz, $\epsilon_\infty = 1.53$, $\gamma = 17.65$ THz and $f = \omega/2\pi$ is the wave frequency, and the slits were then coated with a three-layer graphene, realizing MLG micro-ribbons embedded in the top metal. Perfectly matched layer (PML) and perfect electric conductor (PEC) boundary conditions were selected in the top and bottom direction along z. The boundary condition along x was periodic. All simulations were performed using a single periodic excitation port. (b) Real (n) and imaginary (k) parts of the three-layer graphene refractive index utilized in the 2D simulations. They were obtained by considering a Drude intraband conductivity for the graphene, having Fermi energy $E_F = 200$ meV and electron mobility $\mu = 1400 V/cm^2s$

In our simulations, the MLG is treated as a transition boundary condition with a complex refractive index from Eqs. S4, S5, calculated using: N=3, n_{sub} ~ 3.6, E_F = 200 meV, and μ = 1406 cm^2/Vs, leading to τ = 23.2 fs and σ_{DC} = 0.55 mS as single layer contributions to the total conductivity. The as-calculated n,k values, shown in Supplementary Fig.1b, are assigned at each air/GaAs interface of the plasmonic/DFB slits. Furthermore, we included a gate dielectric layer between the MLG and the top contact of the DFB resonator. The introduction of the gate provides an efficient tool to alter the MLG Fermi energy, E_F, which potentially could change the conversion efficiency (CE) associated with an intracavity field induced frequency up-conversion. For the dielectric, we inserted a ~100-nm-thick HfO_2 layer (refractive index real part n_{HfO2} = 4.2, imaginary part k_{HfO2} = 0) between the doped layer and the top metal grating. This enables a field-effect control of the free carriers in the MLG film using a gate voltage applied between the top metal and the n-doped layer.

S2. Numerical model for third-harmonic generation in the electrically pumped laser structure

We performed electromagnetic simulations to predict the resonances of the designed laser cavity, and to compute the predicted CE of THG, by solving Maxwell's equations at three times ($3\nu_0$) the fundamental frequency ν_0 of the core DFB QCL. To achieve this, we mimicked the external (from the top) excitation of the designed QCL structure with the help of a periodic input port, keeping the impinging power density as a free parameter – this is the most critical parameter for estimating the conversion efficiency (see Supplementary Fig.2a). To extract quantitatively the CE, defined as the ratio between the power delivered at $3\nu_0$ to the input power, the input port power was set to match the power density range illuminating the plasmonic grating intracavity during lasing. We considered the maximum power (≥~120 mW) delivered by surface-emitting 2nd order DFB THz QCLs fabricated on the same active region with identical layout and dimensions. Then, to estimate the actual intracavity power, we normalized this by considering the laser internal quantum efficiency ~40%[6], thus obtaining an intracavity power (P_{in}) ~ 300 mW. To calculate the electric field, we first considered the intracavity power density, I_{in} (W/m^2), irradiating the plasmonic ribbon plane, corresponding to an equivalent electric field: $E = \sqrt{Z_0 I_{in}}$, where Z_0 = 377 Ω is the vacuum impedance. I_{in} is set in agreement with the relation, $I_{in} = \frac{P_{in}}{A}$, under the reasonable assumption that the light-outcoupling occurs uniquely through the central slit (total area A= ~ 200 μm^2) of the entire DFB grating, as deduced by the far-field profile acquired experimentally (see Fig.3e).

To estimate the conversion efficiency (CE) associated with the frequency up-conversion process in the engineered double-grating laser, we implemented a refined numerical model following

the approach proposed in Ref[7]. This method allows one to extract the THG CE as an output parameter directly from simulations, by setting up the equations for the electric field generated by the 3rd-order frequency up conversion process in the software module. The simulation layout comprises the unit cell for the resonator defined in Supplementary Fig.1. The method assumes that MLG is a surface current generator, accounting for both the linear (1st-order) and nonlinear (3rd-order) contributions. Such a current generator is first used to generate the linear electric field at the fundamental harmonic, E_{FH}, relying on the linear conductivity:

$$J_0 = \sigma_0 E_{FH} \tag{S7}$$

Then, during a second simulation iteration, the computation generates the third harmonic field E_{TH}, relying on the field-dependent third order conductivity from the following equations:

$$J_{tot} = \sigma_0 E_{TH} + J_3 \tag{S8}$$

where

$$J_3(\nu) = \sigma_3(\nu)[|E_{FH}(\nu)|^3] \tag{S9}$$

The non-linear interaction between MLG Dirac carriers and an intense driving THz electromagnetic field is governed by the thermodynamic heating/cooling of the free carriers[8,9]. In the THz range, the intraband absorption of a high-power optical beam leads to a non-equilibrium state with an excess distribution of carriers at the energy of the optical pump. The ultrafast (~20 fs) carrier-carrier scattering[10] drives the initial energy redistribution, bringing the system into a non-equilibrium state with electrons sharing a hot-electron temperature[11], before the system relaxes back to an equilibrium state.[10] When the optical excitation pulse (~μs in the present case) varies on a timescale much longer than the internal relaxation time of the MLG (~ps)[12], the Dirac carriers undergo a ~∞ set of heating/cooling cycles, with a steady excitation state at an electronic temperature of T_e being reached[13], where:

$$T_e = T_{sub} + \frac{I_{in}\tau_{cool}}{C_e} \tag{S10}$$

with T_{sub} being the temperature at equilibrium, I_{in} the excitation power intensity, in units of Wcm^{-2}, and τ_{cool} the cooling time. C_e, the graphene heat capacitance, which for moderate Fermi energies ($E_F >> K_B T_e$) can be expressed as[13]:

$$C_{e,doped} = \frac{2\pi E_F}{3(\hbar v_F)^2} k_B^2 T_e \tag{S11}$$

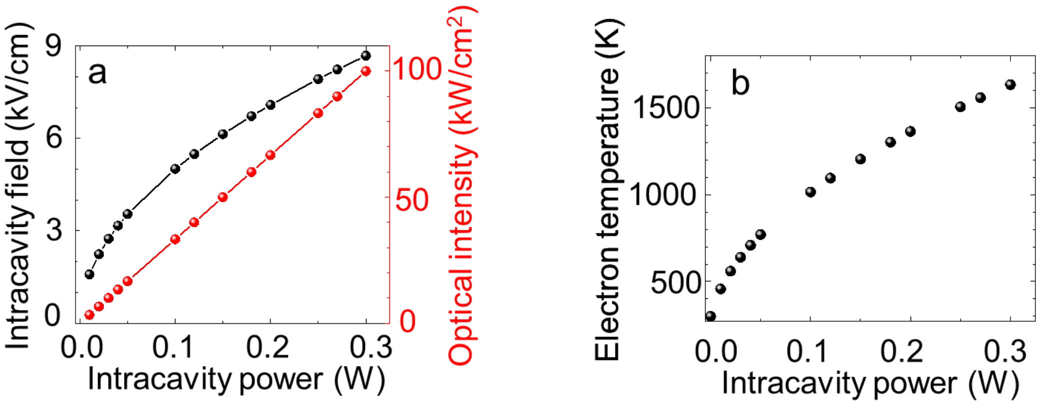

Supplementary Figure 2: (a) Electric field (E, black dots) and optical intensity (I_{in}, red dots) of the incident excitation port as a function of intracavity power (P_{in}) calculated as $E=\sqrt{2I_{in}Z_{vac}}$, where $I_{in}=P_{in}/(W_{DFB}\times L_{DFB})$, is the intracavity power density, concentrated in the area $W_{DFB}\times L_{DFB}$ of one single slit of the DFB grating and Z_0=377 Ω is the vacuum impedance. The width and length of the slit were set to beW_{DFB} = 2.5 µm, and L_{DFB} (120 µm), respectively. (b) Hot electron temperature of Dirac fermions in the MLG ribbons as a function of intracavity power, calculated according to the method of section S2.

From previous reports of SLG on quartz, we assume τ_{cool}=2.5 ps[14,15], and by combining Eqs. S10 and S11, and setting E_F =200 meV, we extract the T_e dependence on the electric optical field, shown in Supplementary Fig. 2b.

The nonlinear term of the conductivity, σ_3, is the Kerr 3rd order conductivity:[16]

$$\sigma_3(\nu) = [\sigma_{Kerr}(\nu)] \qquad \text{(S12)}$$

whose numerical expressions is[16]:

$$\sigma_{Kerr}(\nu) = \frac{i9e^6 v_F}{4\pi\hbar^4}\frac{D_{he}}{(2\pi\nu+i\Gamma_{he})(-2\pi\nu+i\Gamma_{he})(4\pi\nu+i\Gamma_{he})} \qquad \text{(S13)}$$

The E_F-dependent parameters D_{he} and Γ_{he} are the hot electron Drude weight and scattering rate, and can be written as:[16]

$$D_{he} = D_0\left[1-\frac{1}{6}\left(\frac{\pi k_B}{E_F}\right)^2 T_e{}^2\right] \qquad \text{(S14)}$$

$$\Gamma_{he} = \Gamma_0\left[1+\frac{1}{6}\left(\frac{\pi k_B}{E_F}\right)^2 T_e{}^2\right] \qquad \text{(S15)}$$

We use the Kerr effect to calculate the field-dependent, higher order terms in the optical conductivity that is mainly responsible of the observation of non-linear effects, dependent on the strength of the driving electric field[3], and for the observed THG signal.

The described model is valid when the intraband absorption is the only available relaxation channel (single band model)[17]. At higher pump power, or lower chemical potential, the electronic temperature may become higher than the Fermi temperature, i.e. $T_e > T_F = E_F/k_B$, and the single band, intraband model is no longer valid. Considering the maximum laser intracavity power P = 0.3 W, this crossover occurs at $E_F\sim$ 200 meV. At lower doping level, the intense power may induce an

increase in the electronic temperature, which is capable of smearing-out the carrier distribution. This can open a channel for interband transitions (two-band model[17]). For interband, multi-photon absorption, the 3rd order non-linear conductivity σ_3, is expressed as[7,18]:

$$\sigma_3 = \frac{i\sigma_0(\hbar v_F e)^2}{48\pi(\hbar\omega)^4} T\left(\frac{\hbar\omega}{2E_F}\right) \quad \text{(S16)}$$

where $\sigma_0 = e^2/4\hbar$, v_F is the Fermi velocity; $T(x) = G(x) - 64G(2x) + 45G(3x)$ is an empirical model equation where $G(x) = ln\frac{1+x}{1-x} + i\pi\theta(|x|-1)$ (θ is the Heaviside step function). The expression in eq. S16 is then used in the simulation module to calculate the conversion efficiency (CE) for $E_F < 200$ meV (see red dots in Fig.2f).

S3. Estimation of the conversion efficiency

To estimate the conversion efficiency (CE) we performed detailed modelling by varying the ribbon size, W_{pl}, in the plasmonic grating, to assess the photonic cavity modes available for lasing, the optical enhancement on the MLG that can be expected, and, ultimately, the TH conversion efficiency.

The reflection spectra in the 2.7 – 3.7 THz range, shown in the 2D map of Supplementary Fig 3a as a function of W_{pl}, unveil the presence of a non-dispersive mode at fixed frequency n_0 = 3.25 THz (peak A), and a second resonance, peak B, which has a frequency that shifts towards peak A at higher micro-ribbon widths, W_{pl}. Upon inspection of the 2D field distribution map (Fig.2a of the main article), peak A is indeed the fundamental DFB mode, set by the central slit and unit cell size, as visible from the modulation of the z-component of the electric field, corresponding to a TEM_0 mode propagating longitudinally. Peak B corresponds to the resonance of the plasmonic grating, the frequency of which is set primarily by the slit size[2].

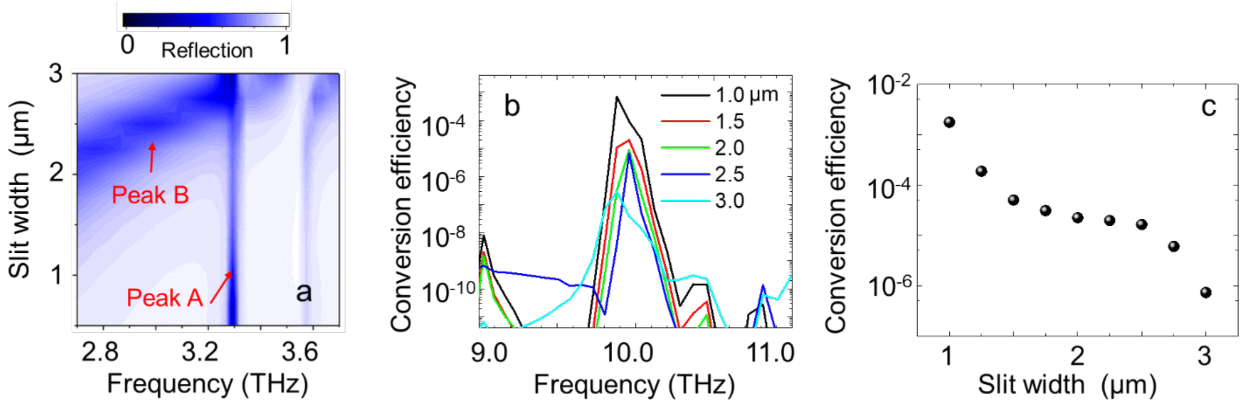

Supplementary Figure 3: (a) Two-dimensional (2D) colour map of the linear reflectance of the graphene-integrated QCL, calculated as a function of frequency and plasmonic slit width. Only the first order terms of the MLG optical conductivity and of the surface current in Eqs. S7-S8 are considered. (b) Third harmonic conversion efficiency as a function of frequency in the graphene-integrated QCL, with different plasmonic slit widths. (c) Third harmonic conversion efficiency at the TH peak frequency as a function of plasmonic slit width. In (a-c), the graphene Fermi energy E_F is set to be 0.2 eV and the intracavity power is 0.3 W.

To promote field enhancement, ideal for an efficient frequency up-conversion, peaks A and B must overlap, which is the condition found at $W_{pl} \geq \sim 2.5$ μm. However, Supplementary Figure 3(b) shows the CE as a function of frequency, calculated with the described numerical model for different values of the slit width for a MLG $E_F \sim 200$ meV. The conversion efficiency is indeed ultimately determined by the field distribution established at the plasmonic ribbon surface. This does not necessarily reach its maximum at the plasmonic grating resonance, but depends more critically on the duty cycle of the grating. A certain degree of field enhancement is indeed always obtained, including out of the plasmonic resonance[19]. The CE value, at the laser fundamental resonance, decreases with increasing W_{pl}, as a direct consequence of the weakening of the electric field enhancement in narrow slits (Supplementary Fig.3c). Indeed, as visible in Supplementary Fig. 4, the field concentrates at the sharp edges of the metal gaps, an effect which is well known in subwavelength metallic structures and exploited in the present device architecture. As a tradeoff, $W_{pl} \sim 2$–2.5 μm is the parameter that was adopted in the fabrication of the engineered lasers.

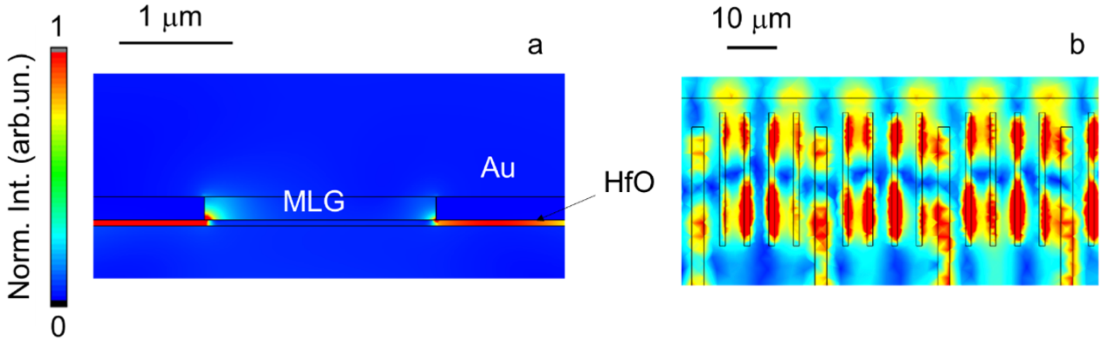

Supplementary Figure 4: Cross (a) and top (b) section views of the electric field amplitude calculated at the fundamental mode frequency (~3.32 THz), for the DFB/plasmonic resonator coated with three layers of graphene. In (a), the field is simulated by combining a surface current density numerical model using 2D simulations with the frequency domain module of COMSOL Multiphysics. Panel (a) shows the magnified view of the electric field in the first ribbon slit with respect to the left of the central DFB aperture shown in Fig.2a (main manuscript). In (b), the electric field is calculated by employing the eigen-frequency solver of the 3D module of COMSOL. Panel (b) shows the magnified view of the electric field in the MLG ribbon array region shown in Fig.1(f) (main manuscript).

S4. Role of the GaAs *Reststrahlenband* on the CE

To evaluate the effect of the *Reststrahlenband* phonons on the TH up-conversion process, we first calculated the total complex refractive index of the GaAs/AlGaAs AR (see Supplementary Fig. 5a), by following the method described in Refs.1,[20].

The GaAs dielectric function ε_{AR} is given by the sum of the Drude term (GaAs free carrier absorption) and the Drude-Lorenz term (TO phonons):

$$\varepsilon_{AR}(\omega) = \varepsilon_{hf}\left(1 - \frac{\omega_p^2}{\omega(\omega + i\tau^{-1})}\right) + \left(\frac{(\varepsilon_{DC} - \varepsilon_{hf})\omega_{TO}^2}{\omega_{TO}^2 - \omega^2 - i\omega\Gamma}\right) \quad \text{(S17)}$$

In Eq.S17, $\omega = 2\pi\nu$, ε_{hf} =10.9 is the high-frequency GaAs dielectric constant, $\omega_p = \sqrt{N_d e^2/\varepsilon_0 \varepsilon_{hf} m^*}$ is the plasma frequency of the free carriers in the AR, calculated by setting m^*=0.067m_e, N_d ~ 4×10^{15} cm^{-3} – the effective doping density of the AR, and τ = 325 fs (the AR scattering time). ε_{DC} =12.9 is the low frequency GaAs dielectric constant, $\omega_{TO} = 2\pi\nu_{TO}$ (ν_{TO} = 8 THz), and $\Gamma = 2\pi \times 0.055$ THz[1,20]. The calculated curves, shown in Supplementary Fig.4a, are then assigned to the AR domain in the simulation module, and the surface current generator calculation is run again. Supplementary Figs.5b-c show the result of the linear response (reflection curve) and nonlinear TH emission, respectively. The presence of the phonon parasitic absorption is visible in the linear response, with a stronger reflection dip at the DFB resonance (see Supplementary Fig.5b). On the other hand, the peak at the TH frequency 3ν_0 is almost unaffected, relying on the fact that the nonlinear emission is taking place in a very confined volume around the plasmonic ribbon surface, and so it is spatially separated from the absorbing medium.

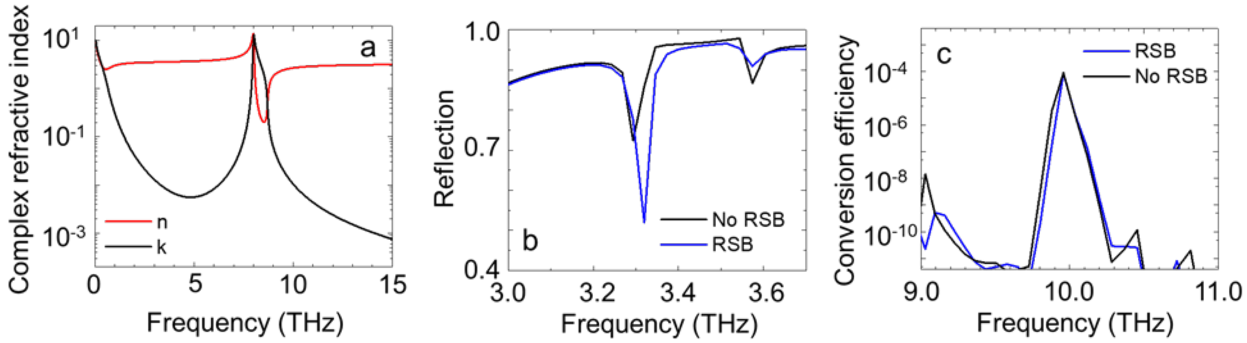

Supplementary Figure 5: (a) Real (n, red) and imaginary (k, black) parts of the complex refractive index of the GaAs/AlGaAs active region (AR), calculated including the TO absorption in the *Reststrahlenband*. (b) Reflection spectra of the MLG integrated QCL device as a function of frequency with (blue) and without (black) the contribution of the *Reststrahlenband* phonons in the AR optical response. The plasmonic slit width is set to 2 um and only the linear part of the MLG optical conductivity is included. (c) Third harmonic conversion efficiency as a function of frequency in the MLG QCL with (blue) and without (black) the *Reststrahlenband* contribution in the AR optical constants. In (b-c) E_F = 0.2 eV and the intracavity power is 0.3 W.

S5. Device fabrication

The core QCL heterostructure is based on a bound to continuum-optical phonon hybrid active region design. The top surface of the 25-µm-thick GaAs/AlGaAs active region was initially coated with Cr/Au and bonded onto a Cr/Au -coated, highly doped GaAs carrier substrate. The host GaAs substrate was then removed through a combination of mechanical lapping and wet etching until an an $Al_{0.5}Ga_{0.5}As$ etch stop layer was reached, which itself was subsequently removed using a 50%-concentration HF etching solution. The fabrication of the 0.2×2.0 mm^2 laser resonators was then undertaken, comprising the patterning of the top metal contact, through a combination of direct laser lithography and metal deposition. A 2nd order distributed feedback Bragg (DFB) grating was defined in the central area of the top contact, using optical lithography, and was followed by the removal of

the 700-nm-thick highly doped-GaAs from the slits, each having size 2×150 μm^2. The slit regions were then coated with a ~30-nm-thick layer of HfO_2. The HfO_2 area was defined through an oxide liftoff, using a combination of electron beam lithography (EBL) and atomic layer deposition (ALD) (performed at room-temperature), soaking the sample in hot acetone to remove the electron-beam resist mask from the areas outside the grating region. The top-plasmonic grating was then aligned to the DFB/plasmonic grating pattern of the DFB slits etched underneath. The resonator fabrication was finally completed by wet-etching of the 25-um-thick active region, using a H_2SO_4/H_2O_2 mixture, ensuring complete removal of the whole heterostructure thickness, and the exposure of the metal ground plane.

The multilayer graphene sample was prepared and transferred using a wet transfer technique: A4-950K ply(methyl-methacrylate) polymer (PMMA) was spin coated at 2,000 r.p.m. onto the surface of a single-layer graphene (SLG) sample (1 cm × 1 cm) grown on copper using chemical-vapour-deposition. After 1 min on a hot plate at 90 °C, the sample was placed in a solution of 1 g of ammonium persulfate and 40 ml of deionized water to etch the copper substrate. Once the copper etching was complete, the PMMA-SLG film was transferred into a beaker of deionized water and then combined with a second copper-graphene square to obtain a bilayer graphene sample. This sample was left to dry completely. The copper of the bilayer graphene was then etched with the same technique.

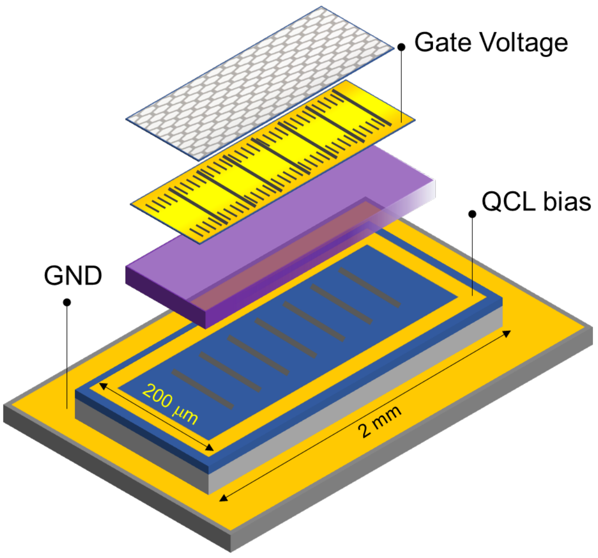

Supplementary Figure 6: Schematic flowchart of the fabrication

This process was repeated until the desired MLG thickness was reached, in our case 3 layers. The multi-stack was then transferred onto the top of the lasers, left to dry, and finally soaked in acetone to clean the surface and remove the PMMA. The MLG was then patterned and removed from the

region outside the DFB/plasmonic grating, using maskless laser lithography and O_2 plasma etching. The fabrication flowchart is schematically sketched in Supplementary Figure 6. The sample carrier substrate was finally lapped down to a thickness of 200 µm, and coated with a 10/100 nm Cr/Au layer to ensure a good thermal heat sink to the copper sample holder for electrically pumped lasing operation, which was carried out in a liquid-helium thermal bath at a temperature in the 15-20 K range.

S6. High power QCL design

The active region used in the present work is based on a high power QCL with an optical bandwidth in the 2.9-3.5 THz range[21,22]. To test the optical performance of the active region, we initially fabricated QCLs having a 'single plasmon' waveguide geometry. To condense the intracavity field into the fundamental mode, 5-nm-thick, 40-µm-wide Ni side-absorbers were introduced on each edge of the ridge to increase the difference in losses between fundamental and higher order transverse modes, and fully suppress the higher-order competing modes. An overlap of 3 µm between each Ni side-absorber and the upper Au over-layer, 150 nm thick, was set by design.

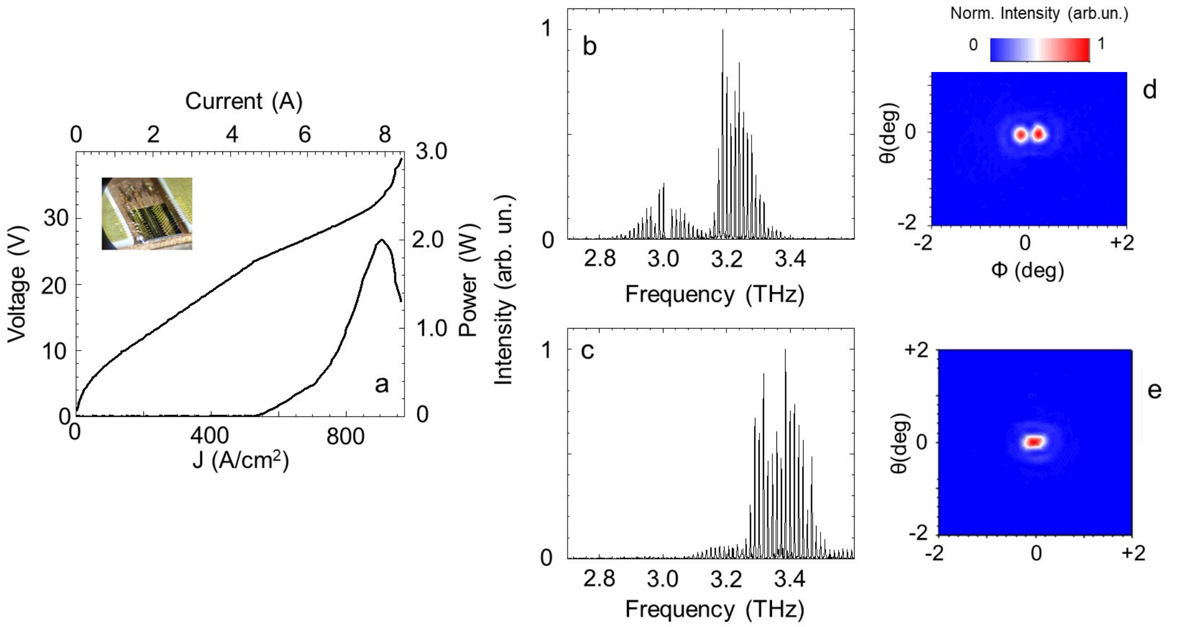

Supplementary Figure 7: (a) Voltage-current density and light-current density (*L-J-V*) characteristics measured while driving the single-plasmon QCL in pulsed mode with a pulse width of 100 ns (1% duty cycle); (b-c) Fourier transform infrared (FTIR) emission spectra, measured in rapid scan mode, at a driving current I=10A (J=825 A/cm^2), corresponding to the peak optical power under vacuum, employing the internal DTGS far-infrared detector and with a spectral resolution of 0.075 cm^{-1}. (d-e) Far-field intensity patterns, collected on the same QCL of (a), while raster scanning a pyro-electric detector onto a spherical surface (radius of ~ 6 cm) cantered on the device.

Supplementary Figs 7 shows (a) the voltage-current-light characteristics, (b-c) the emission spectra and (d-e) the far field intensity pattern obtained for a single plasmon QCL realized with (S7c/e), and

without (S7b/d), the Ni side aborbers[23]. An effect of the loss induced by the Ni side absorbers is to attenuate the low frequency modes and for the QCL bandwidth to become centered around 3.35 THz.

S7. Micro-Raman Spectroscopy on the MLG

Raman spectroscopy was used to monitor the MLG quality and doping, following transfer onto the QCL devices.[24,25] Spectra (Supplementary Fig. 8) were acquired with a Renishaw Invia, at 532 nm using a 50 × objective, with an optical power density <0.3 mW/μm^2 to prevent sample heating, We found peaks at Pos (G) =1586 ± 4 cm^{-1} and Pos (2D) = 2698 ± 8 cm^{-1} from a statistical analysis of five spectra at different points on the sample surface. The 2D and G peaks are single Lorentzians with full-width-at-half-maximum FWHM (2D) = 53 ± 10 cm^{-1}, and FWHM (G) = 32 ± 1 cm^{-1}. The 2D to G peak intensity and area ratios are I(2D)/I(G) = 2.4 ± 1.3 and A(2D)/A(G) = 2.5 ± 1.3, corresponding to p doping ~ 200meV.[26] I(D)/I(G) = 1.2 ± 0.5, indicating the presence of Raman active defects.

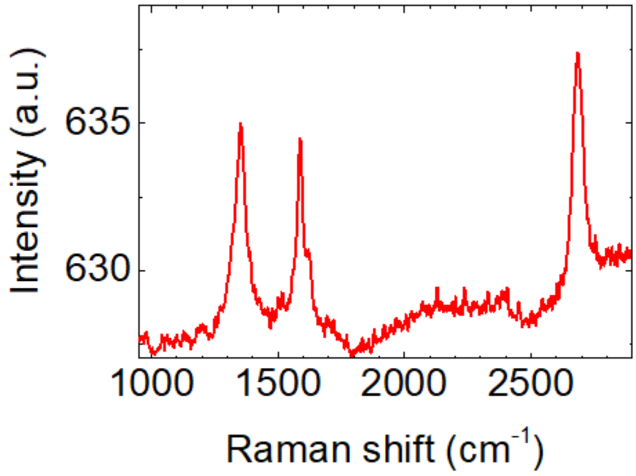

Supplementary Figure 8: Typical micro-Raman spectrum acquired on the three-layer MLG deposited on top of the plasmonic grating QCL, with a 532 nm optical excitation and 50× magnification.

S8. Summary of QCL performances

Table I summarizes the results obtained on a set of four additional devices, each nominally identical to the ones shown in the main article and measured following the same experimental procedure described in the manuscript, i.e. rapid scan FTIR acquisition of the main peak position ν_0, and filtered FTIR step scan acquisition for the 3ν_0 peak.

The collected data on the additional samples show that ν_0 ~3.25-3.35 THz, in broad agreement with the DFB grating design. In all cases ν_0 red-shifts when V_G is approaching the minimum conductivity point ($E_F \approx$ 50 meV) with a tuning coefficient of ~10-20 GHz/V. A well-defined peak emerges above the noise level (S/N≥10) at 3ν_0, the THG frequency, for gate voltages approaching the CNP, in perfect agreement with the results reported in the main text. We estimate a third harmonic

conversion efficiency ranging from ~ (1. to 3.0) $\times 10^{-5}$, extracted considering the total amplitude signal ratio between the filtered and the intracavity power. This latter quantity was estimated by considering the non-filtered signal amplitude, normalized by considering the laser internal quantum efficiency ~40%[6], and an empirical ~50% absorption in the light outcoupling through the MLG. The maximum CE retrieved at the gate voltage corresponding to the minimum Fermi energy (E_F), and the range of investigated E_F, are also listed in Table S1.

ν	*3 ν₀*	*Conversion Efficiency*	*MLG Fermi energy*
3.270 THz	9.81 THz	$(1.9\pm0.5)\text{x}10^{-5}$	50-300 meV
3.275 THz	9.82 THz	$(1.25\pm0.5)\text{x}10^{-5}$	50-250 meV
3.23 THz	9.65 THz	$(3.0\pm0.3)\text{x}10^{-5}$	50-210 meV
3.37 THz*	9.65 THz	$(1.7\pm0.5)\text{x}10^{-5}$	50-210 meV
3.25 THz**	9.65 THz	$(1.03\pm0.8)\text{x}10^{-5}$	50-300 meV
3.26 THz	9.78 THz	$(2.1\pm0.5)\text{x}10^{-5}$	50-300 meV
3.26 THz***,+	9.78 THz	$(5.4\pm0.5)\text{x}10^{-5}$	50-300 meV

Supporting Table I: Maximum conversion efficiencies
*Device shown in Fig.4a,d,h of the main text
**Device shown in Fig.4b,e of the main text
***Device shown in Fig.4c,f,h of the main text
+ Device fabricated with the optimized procedure

The performances of a prototypical device, fabricated with the new production procedure, is listed in the last row of Table I (highlighted in grey). Similar CE values have been extracted for the entire set of lasers belonging to the same fabrication run.

S9. 2nd order DFB quantum cascade laser

The 2nd order DFB grating QCL is the benchmark laser design used to conceive the DFB/plasmonic resonator concept. In Supplementary Fig. 9 we show the LIV curve obtained on a top-emitting prototypical DFB pristine laser, i.e. without the integration of graphene, fabricated employing the high-power AR material of section S6, with the same size (2×0.2 mm^2) and grating geometry (DFB grating period 25.5 μm, slit width 2 μm).

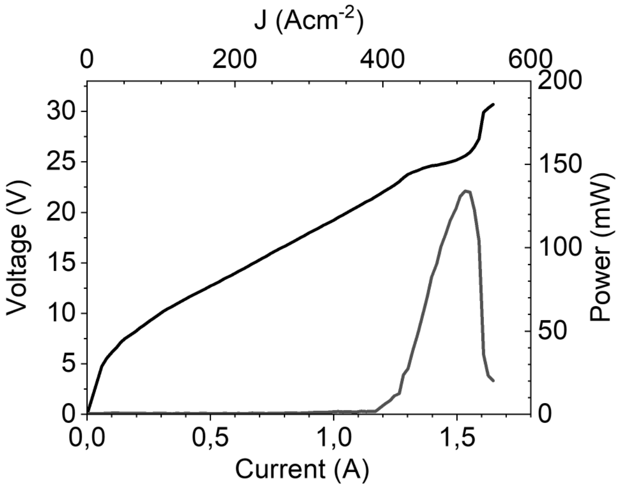

Supplementary Figure 9: Voltage-current density and light-current density (L-J-V) characteristics measured at 20K while driving the 2nd order DFB QCL in pulsed mode with a pulse width of 1 µs (duty cycle 1%), repetition rate of 10 kHz. The peak power (right axis) is acquired using a calibrated power meter (Thomas Keating).

S10 High-pass Ta filter transmittance

Supplementary Figure 10 shows the normalized transmittance of the 2-mm-thick Ta high-pass filter (Crystan limited) used to separate the up-converted terms in the spectra from the fundamental harmonic. The cutoff frequency (υ_{co}) is defined as the frequency at which the transmittance is attenuated by a $\sqrt{2}$ factor, $T(\upsilon_{co}) = \sqrt{2} \times T_{max}$. Then υ_{co} = 7 THz from our data.

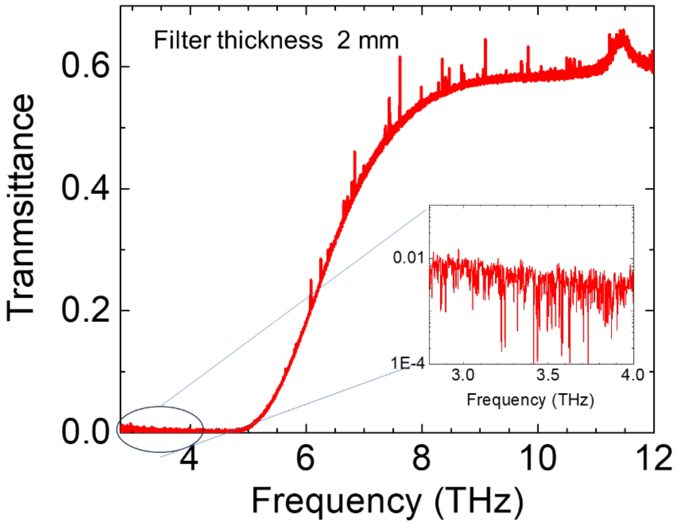

Supplementary Figure 10: Transmittance of the 2-mm-thick thallium high-pass filter used for suppression of the QCL fundamental mode; data was acquired with a spectral resolution of 0.15 cm-1. The trace was obtained by normalizing the curve acquired in transmission mode through the filter, with the internal Globar source of the FTIR spectrometer under vacuum as a reference. The inset shows the 3-4 THz frequency range. At 4 THz, the transmittance was <0.7%.

S11. Experimental procedure to extract the collection efficiency

The value of the conversion efficiency (CE) is extracted experimentally through the equation:

$$\mathrm{CE} = \frac{I_{TGH}}{I_{IFH}} = \frac{I_{TGH}}{I_{FH} \times I_{QE} \times A_{MLG}} \quad \text{(S18)}$$

where I_{THG} is the optical intensity of the third harmonic signal, and I_{IFH} is the intensity of the mode at the fundamental frequency, converted to its intracavity value as follows: $I_{IFH} = I_{FH} \times I_{QE} \times A_{MLG}$, where I_{FH} is the fundamental mode optical power emitted by the DFB QCL, I_{QE} is the internal quantum efficiency, and A_{MLG} is the graphene multi-stack absorption coefficient. We set I_{QE}=1.6 (quantum efficiency 60%), and $A_{MLG}=0.5$, following Refs.[6], and [14,27], respectively.

In eq. S18, the ratio I_{THG}/I_{FH} is retrieved from the interferograms acquired in step scan, respectively without (Supplementary Fig.11a) and with (Supplementary Fig.11b) the Ta filter. In fact, the ratio I_{THG}/I_{FH} is proportional to the ratio of the average amplitudes of the sinusoidal-like traces, at the beginning of the scan, i.e. closer to the FTIR zero-path difference, where the amplitude of the sinusoidal oscillation is maximum. The amplitudes are then normalized by the lock-in sensitivity setting for each trace, as is explained further in the caption of Supplementary Fig.11.

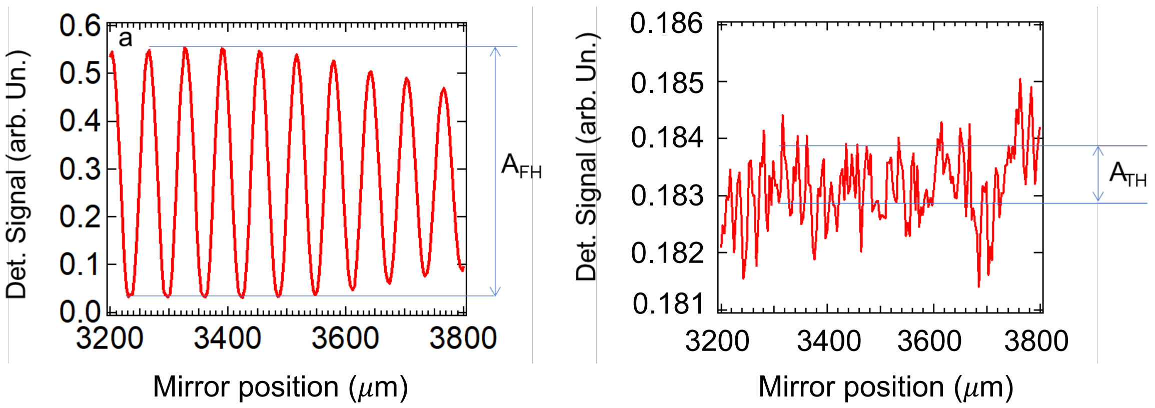

Supplementary Figure 11: a-b) Illustrative interferogram traces from the devices reported in Figs.4a,c of the main text, acquired in step-scan mode, (a) without the Ta filter (fundamental mode), using a lock-in sensitivity of S_{FH}=300 mV, and (b) with the Ta filter (TH mode) and lock-in sensitivity S_{TH}=10 mV; both sets of data were measured at a gate voltage V_G=+3V (black curve of fig.4c). The arrows and horizontal lines highlight the average amplitude values extracted from the data, and which were used to estimate the respective optical intensities, namely A_{FH}=0.55 and A_{TH}=0.00097. By accounting for the lock-in sensitivities, the ratio I_{TH}/I_{FH} is then 0.58×10^{-4}, and the final value of the CE, considering the intracavity power and the MLG absorption is ~1.65×10^{-5}.

S12 Double-grating surface emitting QCL without MLG

To corroborate our claim that HG arises from the MLG, we have performed an identical fabrication run, omitting the transfer of the three-layer graphene onto the top contact of the DFB/plasmonic laser (see schematic in Supplementary Fig.12a), and collected FTIR spectra under the same experimental conditions of Figs 4a-f. (see Supplementary Fig. 12b-e).

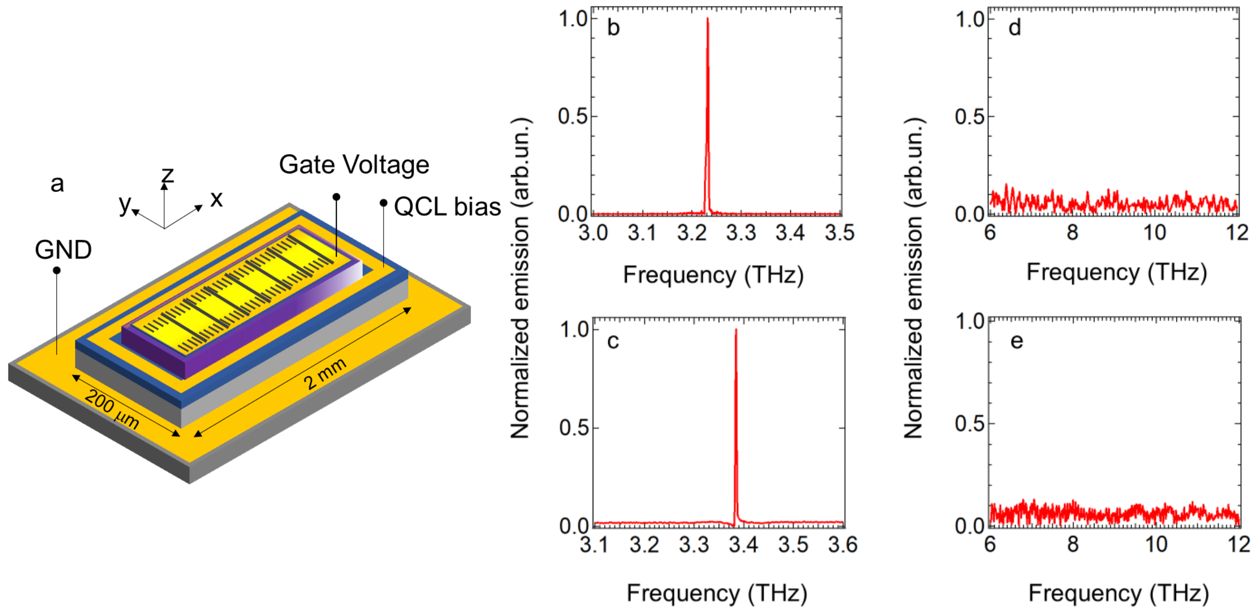

Supplementary Figure 12 (a) Schematic representation of the hybrid plasmonic quantum cascade laser (QCL) design, featuring a surface emitting, 2nd order distributed feedback (DFB) grating fabricated within the top-contact of a double-metal waveguide resonator, with emission centred at ~3.3 THz. A bare plasmonic grating is embedded into the top contact, i.e. without the MLG covering the area patterned with the ribbons and the DFB slits. b-e) Under vacuum FTIR emission spectra of two prototypical devices, with central DFB frequency shifted by about 0.1 THz (b-c, d-e) measured at a driving current corresponding to the peak optical power emission. (b,c) were taken in rapid scan mode with a 0.075 cm^{-1} spectral resolution; (d,e) were taken in a step scan mode with a 1 cm^{-1} spectral resolution, after filtering out the fundamental mode with a Ta filter, using the same procedure described for Fig.4. (c-e).

Whilst the fabricated QCLs show light emission in the same frequency range as the MLG-integrated devices, as visible from the rapid scan un-filtered spectra shown in Supplementary Figs. 12b,c, the filtered (thallium filter) step-scan spectra collected under identical experimental conditions did not show any signal at the third harmonic frequency (Supplementary Figs. 12d,e).

S13. Second harmonic generation in MLG-integrated QCLs

The investigated samples did not show any second harmonic (SH) signal, as expected due to the centro-symmetrical nature of the hexagonal SLG structure. An anisotropic SH signal could in principle arise in SLG on SiO_2[28], or in graphite-like multilayer graphene sample[29–32], if the crystal orientation of the SLG hexagonal structure with respect to the substrate crystal fulfils the conditions for dipolar symmetry breaking[33]. However, in the polycrystalline SLG used in this work to realize the three-layer stack, comprising single crystalline domains of a few micrometres size and random crystalline orientations, the latter condition is not satisfied. Furthermore, the MLG is realized by stacking one SLG on top of another single graphene layer, and so the control of the mutual orientation of each single-crystal domain, that could be responsible of possible symmetry breaking effects[33] with respect to the adjacent top and bottom planes, cannot be achieved. SH generation in graphene can also

be realized with tens of kV/cm pump fields in a free electron laser (FEL) [34] which drives carriers to the impact ionization region that induces the symmetry breaking needed to generate the SH. An analogue mechanism could be indeed driven by the field penetration from the dielectric layer to the graphene film. However, in the present experiment, we estimate electric fields to be < 9 kV/cm, i.e. significantly lower than the carrier density ($\gtrsim 10^{18}$ cm-3)/field-driven velocity (field $\gtrsim$25 kV/cm) thresholds[34].

Supplementary Figure 13 shows the comparison between the step-scan spectra of Figs 4d,e, measured on the samples with the MLG stack on the top contact, and the two traces shown in Supplementary S12d and S12e, measured on the bare samples (no MLG stack integration), plotted with the same vertical scale, in the 6-7.5 THz range. In the frequency range corresponding to the second harmonic frequency, highlighted by the vertical dotted lines for the MLG-integrated laser of Fig.4d,e, the signal to noise ratio of the different curves is comparable, and no visible peaks are observed above the background noise.

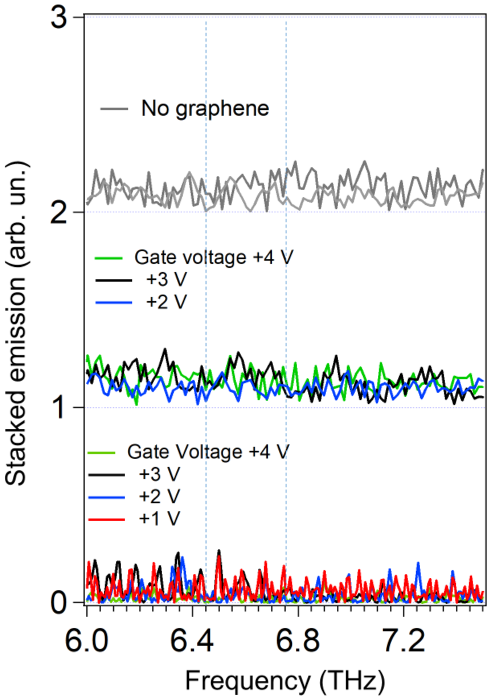

Supplementary Figure 13: Stacked emission spectra acquired in step-scan mode after filtering out the fundamental mode with a 2 mm thick Ta filter, with spectral resolution of 1 cm^{-1}, in the frequency range of interest for the SHG of the fabricated QCLs. From top to bottom: grey lines, spectra acquired from two identical bare QCLs (grey lines, same data shown in S12d,e). middle: spectra retrieved from the MLG-integrated QCL of fig.4d,e of the main article, acquired at gate voltage +4 V (green), +3 V (black) and +2 V(blue). bottom: spectra retrieved from the MLG-integrated QCL of fig.4a,b of the main article, acquired at gate voltage +4 V (green), +3 V (black), +2 V(blue), and +1 V(red).

S14. - Lasers fabricated without HfO_2

Supplementary Figure 4a shows the high intensity field concentration in the thin HfO_2 layer that results from the lossless dielectric layer (real part of the refractive index n_{HfO2}~4.4), which is

sandwiched between the top metal contact and the highly conductive n-doped layer underneath, where the electric field is very unlikely to penetrate. The presence of such a thin dielectric spacer, which is essential to tune the Fermi level in graphene and, consequently, to maximize the conversion efficiency, is indeed also beneficial because it allows high field concentrations in close proximity of the plasmonic graphene grating, where the non-linear frequency up-conversion process takes place.

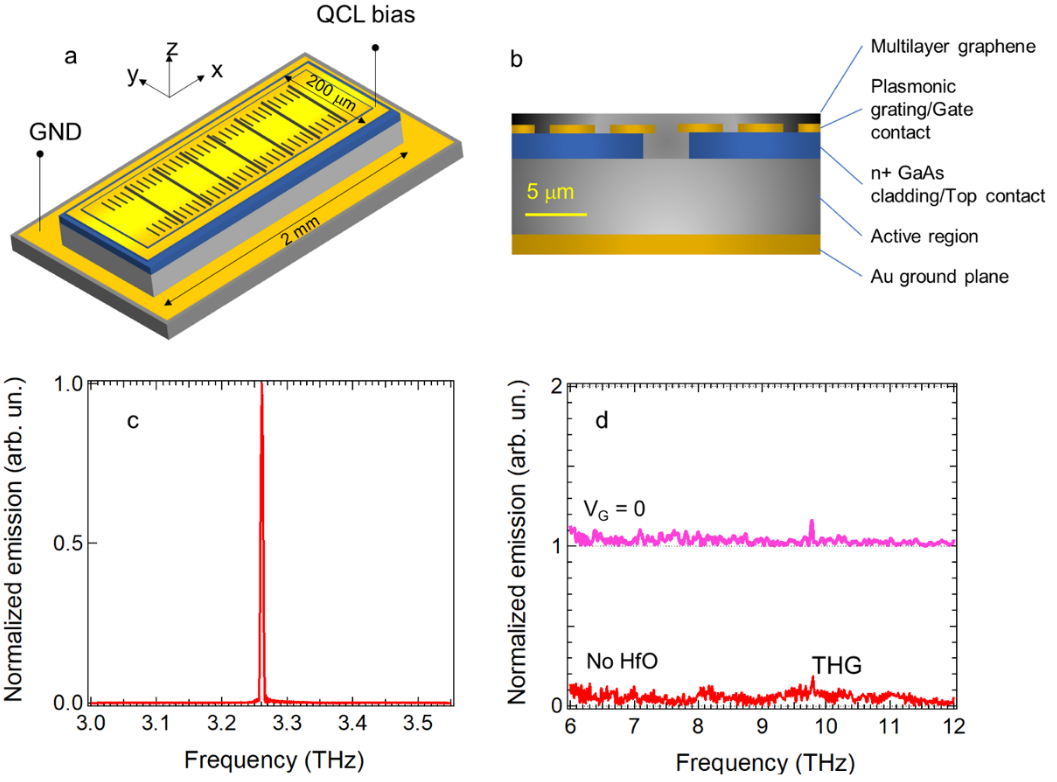

Supplementary Figure 14.(a,b) Schematic side (a) and cross-section (b) view of a QCL fabricated without the thin HfO layer, but with the same DFB/plasmonic grating embedded in the top contact. (c) FTIR emission spectra (under vacuum) of a prototypical device with the design shown in (a,b), measured at a driving current corresponding to the peak optical power emission, in rapid scan mode with a 0.075 cm^{-1} spectral resolution. (d) FTIR emission spectra, measured in step scan mode with a 1 cm^{-1} spectral resolution, after filtering out the fundamental mode with a Ta filter, using the same procedure described for Fig.4.(d-f), for the device of panel S14c (red trace), and for the device of Fig.4f, driven at V_G=0 V (pink). The y-axis is normalized with respect to the highest intensity peak, measured at the third harmonic frequency (green trace in Fig.4f).

To exclude any possible frequency up-conversion process originating from the thin HfO layer itself, we have tested an identical laser (i.e. the same DFB/plasmonic grating on the top) to the one shown in Fig.4c, but fabricated without the HfO_2 layer between the doped layer and the top metal (see Supplementary Fig.14a,b). A small peak at the third harmonic frequency is still seen at zero gate voltage, with comparable intensity (see Supplementary Fig.14c,d) to those measured in the devices already presented in the present work, confirming that only graphene is responsible for the observed effect THG. We note that, as in Supplementary Figure 13, there is no evidence of any peak at the second harmonic frequency.

References

1. Janipour, M., Misirlioglu, I. B. & Sendur, K. A Theoretical Treatment of THz Resonances in Semiconductor GaAs p–n Junctions. *Materials* vol. 12 (2019).
2. Jadidi, M. M. *et al.* Tunable Terahertz Hybrid Metal–Graphene Plasmons. *Nano Lett.* **15**, 7099–7104 (2015).
3. Di Gaspare, A. *et al.* Electrically Tunable Nonlinearity at 3.2 Terahertz in Single-Layer Graphene. *ACS Photonics* (2023) doi:10.1021/acsphotonics.3c00543.
4. Horng, J. *et al.* Drude conductivity of Dirac fermions in graphene. *Phys. Rev. B* **83**, 165113 (2011).
5. Mak, K. F. *et al.* Measurement of the Optical Conductivity of Graphene. *Phys. Rev. Lett.* **101**, 196405 (2008).
6. Vitiello, M. S. *et al.* Probing quantum efficiency by laser-induced hot-electron cooling. *Appl. Phys. Lett.* **94**, 21115 (2009).
7. Guo, T., Jin, B. & Argyropoulos, C. Hybrid Graphene-Plasmonic Gratings to Achieve Enhanced Nonlinear Effects at Terahertz Frequencies. *Phys. Rev. Appl.* **11**, 24050 (2019).
8. Hafez, H. A. *et al.* Terahertz Nonlinear Optics of Graphene: From Saturable Absorption to High-Harmonics Generation. *Adv. Opt. Mater.* **8**, 1900771 (2020).
9. Soavi, G. *et al.* Hot Electrons Modulation of Third-Harmonic Generation in Graphene. *ACS Photonics* **6**, 2841–2849 (2019).
10. Tomadin, A., Brida, D., Cerullo, G., Ferrari, A. C. & Polini, M. Nonequilibrium dynamics of photoexcited electrons in graphene: Collinear scattering, Auger processes, and the impact of screening. *Phys. Rev. B* **88**, 35430 (2013).
11. Soavi, G. *et al.* Broadband, electrically tunable third-harmonic generation in graphene. *Nat. Nanotechnol.* **13**, 583–588 (2018).
12. Tomadin, A. *et al.* The ultrafast dynamics and conductivity of photoexcited graphene at different Fermi energies. *Sci. Adv.* **4**, eaar5313 (2023).
13. Massicotte, M., Soavi, G., Principi, A. & Tielrooij, K.-J. Hot carriers in graphene – fundamentals and applications. *Nanoscale* **13**, 8376–8411 (2021).
14. Dawlaty, J. M. *et al.* Measurement of the optical absorption spectra of epitaxial graphene from terahertz to visible. *Appl. Phys. Lett.* **93**, 131905 (2008).
15. Brida, D. *et al.* Ultrafast collinear scattering and carrier multiplication in graphene. *Nat. Commun.* **4**, 1987 (2013).
16. Han, J. W. *et al.* Plasmonic Terahertz Nonlinearity in Graphene Disks. *Adv. Photonics Res.* **3**, 2100218 (2022).
17. Kovalev, S. *et al.* Electrical tunability of terahertz nonlinearity in graphene. *Sci. Adv.* **7**, eabf9809 (2023).
18. Cheng, J. L., Vermeulen, N. & Sipe, J. E. Third order optical nonlinearity of graphene. *New J. Phys.*

16, 53014 (2014).

19. Deinert, J.-C. *et al.* Grating-Graphene Metamaterial as a Platform for Terahertz Nonlinear Photonics. *ACS Nano* **15**, 1145–1154 (2021).
20. Manceau, J.-M., Zanotto, S., Sagnes, I., Beaudoin, G. & Colombelli, R. Optical critical coupling into highly confining metal-insulator-metal resonators. *Appl. Phys. Lett.* **103**, 91110 (2013).
21. Song, C. *et al.* High-power density, single plasmon, terahertz quantum cascade lasers via transverse mode control. *Appl. Phys. Lett.* **122**, 121108 (2023).
22. Li, L. *et al.* Terahertz quantum cascade lasers with >1 W output powers. *Electron. Lett.* **50**, 309–311 (2014).
23. Garrasi, K. *et al.* High Dynamic Range, Heterogeneous, Terahertz Quantum Cascade Lasers Featuring Thermally Tunable Frequency Comb Operation over a Broad Current Range. *ACS Photonics* **6**, 73–78 (2019).
24. Ferrari, A. C. *et al.* Raman Spectrum of Graphene and Graphene Layers. *Phys. Rev. Lett.* **97**, 187401 (2006).
25. Ferrari, A. C. & Basko, D. M. Raman spectroscopy as a versatile tool for studying the properties of graphene. *Nat. Nanotechnol. 2013 84* **8**, 235–246 (2013).
26. Das, A. *et al.* Monitoring dopants by Raman scattering in an electrochemically top-gated graphene transistor. *Nat. Nanotechnol.* **3**, 210–215 (2008).
27. Di Gaspare, A. *et al.* Self-Induced Mode-Locking in Electrically Pumped Far-Infrared Random Lasers. *Adv. Sci.* **10**, 2206824 (2023).
28. Dean, J. J. & van Driel, H. M. Graphene and few-layer graphite probed by second-harmonic generation: Theory and experiment. *Phys. Rev. B* **82**, 125411 (2010).
29. Han, X. *et al.* Chemical Potential Characterization of Symmetry-Breaking Phases in a Rhombohedral Trilayer Graphene. *Nano Lett.* **23**, 6875–6882 (2023).
30. He, M. *et al.* Symmetry breaking in twisted double bilayer graphene. *Nat. Phys.* **17**, 26–30 (2021).
31. Dai, Y. Y. *et al.* Symmetry breaking induced excitations of dark plasmonic modes in multilayer graphene ribbons. *Opt. Express* **24**, 20021–20028 (2016).
32. Lee, Y. *et al.* Competition between spontaneous symmetry breaking and single-particle gaps in trilayer graphene. *Nat. Commun.* **5**, 5656 (2014).
33. Zou, K., Zhang, F., Clapp, C., MacDonald, A. H. & Zhu, J. Transport Studies of Dual-Gated ABC and ABA Trilayer Graphene: Band Gap Opening and Band Structure Tuning in Very Large Perpendicular Electric Fields. *Nano Lett.* **13**, 369–373 (2013).
34. Wen, Y. *et al.* A universal route to efficient non-linear response via Thomson scattering in linear solids. *Natl. Sci. Rev.* **10**, nwad136 (2023).
