# Supplementary material for: Electrically driven heterostructured far-infrared wire lasers with integrated graphene plasmons
Source: Nat Nanotechnol. 2025 Oct 30;20(11):1611–7. doi: 10.1038/s41565-025-02005-z (PMC12623234; doi:10.1038/s41565-025-02005-z)
Supplement: Supplementary file 2 — Reporting Summary [file 41565_2025_2005_MOESM2_ESM.pdf]

nature research

Corresponding author(s): Miriam Serena Vitiello NNANO-25020739B

☐ Initial submission ☐ Revised version ☒ Final submission

Lasing Reporting Summary

Nature Research wishes to improve the reproducibility of the work that we publish. This form is intended for publication with all accepted papers reporting claims of lasing and provides structure for consistency and transparency in reporting. Some list items might not apply to an individual manuscript, but all fields must be completed for clarity.

For further information on Nature Research policies, including our data availability policy, see Authors & Referees.

Experimental design

Please check: are the following details reported in the manuscript?

1. Threshold

Item	Yes/No	Details
Plots of device output power versus pump power over a wide range of values indicating a clear threshold	☒ Yes ☐ No	Emitted power VS input current of the electrically pumpud lasers

2. Linewidth narrowing

Item	Yes/No	Details
Plots of spectral power density for the emission at pump powers below, around, and above the lasing threshold, indicating a clear linewidth narrowing at threshold	☒ Yes ☐ No	Figures 4 how the emission in the spectral ranges of interest collected with an FTIR spectrometer. This technique does not allow to estimated the lasing linewidth.
Resolution of the spectrometer used to make spectral measurements	☒ Yes ☐ No	Caption of Fig.4 includes the details

3. Coherent emission

Item	Yes/No	Details
Measurements of the coherence and/or polarization of the emission	☒ Yes ☐ No	Lasing reported in a quantum cascade heterisctructure. Ineherent Coherent emission

4. Beam spatial profile

Item	Yes/No	Details
Image and/or measurement of the spatial shape and profile of the emission, showing a well-defined beam above threshold	☒ Yes ☐ No	Shown in Figures 3d,e

5. Operating conditions

Item	Yes/No	Details
Description of the laser and pumping conditions *Continuous-wave, pulsed, temperature of operation*	☒ Yes ☐ No	All the details are given in manuscript, figure captions and supporting information
Threshold values provided as density values (e.g. W cm^{-2} or J cm^{-2}) taking into account the area of the device	☒ Yes ☐ No	Included in Figure 3, and in the Supplementary Information

6. Alternative explanations

Item	Yes/No	Details
Reasoning as to why alternative explanations have been ruled out as responsible for the emission characteristics *e.g. amplified spontaneous, directional scattering; modification of fluorescence spectrum by the cavity*	☒ Yes ☐ No	Modelling and data to support frequency upconversion carafully reported

7. Theoretical analysis

Item	Yes/No	Details
Theoretical analysis that ensures that the experimental values measured are realistic and reasonable *e.g. laser threshold, linewidth, cavity gain-loss, efficiency*	☒ Yes ☐ No	Detailed modelling in the manuscript and Supporting Information file

8. Statistics

Item	Yes/No	Details
Number of devices fabricated and tested	☒ Yes ☐ No	Supporting Information sections dedicated to the statistical analysis of the various devices tested and the number is propertly reported.
Statistical analysis of the device performance and lifetime (time to failure)	☒ Yes ☐ No	A statistical analysis of the main figures of merit is reported in the Supporting Information<br>The presented devices are extremelvery stable. Hence the inherent device lifetime (years) is much longer than a set of repeated experimental campaigns

Further reading

We also suggest that authors read the following literature, which describes the important principles and signatures of laser emission and discusses some of the common mistakes that can occur during laser characterization.

1. Samuel I.D.W., Namdas, E.B. & Turnbull, G.A. How to recognize lasing. *Nat. Photon.* **3**, 546-549 (2009).
2. Siegmann, A.E. *Lasers*. (University Science Books, 1990)
3. Svelto, O. *Principles of Lasers*. 5th edn. (Springer 2010)
4. Blood, P. *Quantum Confined Laser Devices: Optical Gain and Recombination in Semiconductors*. (Oxford Univ. Press, 2015)
5. Koxlov, V.G. *et al.* Laser action in organic semiconductor waveguide and double-heterostructure devices. *Nature* **389**, 362-364 (1997).
